# From the discovery of field ionization to field desorption and liquid injection field desorption/ionization-mass spectrometry—A journey from principles and applications to a glimpse into the future

**DOI:** 10.1177/1469066720939399

**Published:** 2020-06-30

**Authors:** Jürgen H Gross

**Affiliations:** Institute of Organic Chemistry, Heidelberg University, Heidelberg, Germany

**Keywords:** Field ionization, field desorption, liquid injection field desorption/ionization, field emitter, strong electric fields, ionization process, molecular ions, cluster ions, soft ionization, desorption ionization

## Abstract

The discovery of the ionizing effect of strong electric fields in the order of volts per Ångstrom in the early 1950s eventually led to the development of field ionization-mass spectrometry (FI-MS). Due to the very low ion currents, and thus, limited by the instrumentation of the 1960s, it took some time for the, by then, new technique to become adopted for analytical applications. In FI-MS, volatile or at least vaporizable samples mainly deliver molecular ions, and consequently, mass spectra showing no or at least minor numbers of fragment ion signals. The next major breakthrough was achieved by overcoming the need to evaporate the analyte prior to ionization. This was accomplished in the early 1970s by simply depositing the samples onto the field emitter and led to field desorption-mass spectrometry (FD-MS). With FD-MS, a desorption ionization method had become available that paved the road to the mass spectral analysis of larger molecules of low to high polarity and even of organic salts. In FD-MS, all of these analytes deliver spectra with no or at least few fragment ion peaks. The last milestone was the development of liquid injection field desorption/ionization (LIFDI) in the early 2000s that allows for sample deposition under the exclusion of atmospheric oxygen and water. In addition to sampling under inert conditions, LIFDI also enables more robust and quicker operation than classical FI-MS and FD-MS procedures. The development and applications of FI, FD, and LIFDI had mutual interference with the mass analyzers that were used in combination with these methods. Vice versa, the demand for using these techniques on other than magnetic sector instruments has effectuated their adaptation to different types of modern mass analyzers. The journey started with magnetic sector instruments, almost skipped quadrupole analyzers, encompassed Fourier transform ion cyclotron resonance (FT-ICR) and orthogonal acceleration time-of-flight (oaTOF) analyzers, and finally arrived at Orbitraps. Even interfaces for continuous-flow LIFDI have been realized. Even though being niche techniques to some degree, one may be confident that FI, FD, and LIFDI have a promising future ahead of them. This Account takes you on the journey from principles and applications of the title methods to a glimpse into the future.

## Introduction

This Account does not provide a comprehensive review covering any aspect of more than six decades of development and applications in field ionization (FI), field desorption (FD), and liquid injection field desorption/ionization (LIFDI) that would comprise several hundreds of references. Rather, it aims at delivering a solid primer for those knowing about the title techniques just from hearsay and at presenting a tutorial for any mass spectrometrist wanting to broaden his/her horizon. Finally, it intends to provide a refresher course on the fundamentals for practitioners and occasional users. Admittedly, the techniques require some knowledge, some getting-used-to, and also a steady hand, but done right, they can deliver highly impressive analytical data. To avoid frustration, practical aspects, dos and don’ts, and general recipes for daily operation are also included. Based on almost three decades of personal experience with FI, FD, and LIFDI and suffering from some fondness for these methods, the author herewith begins the Account.

## Historical sketch

**Figure 1.**
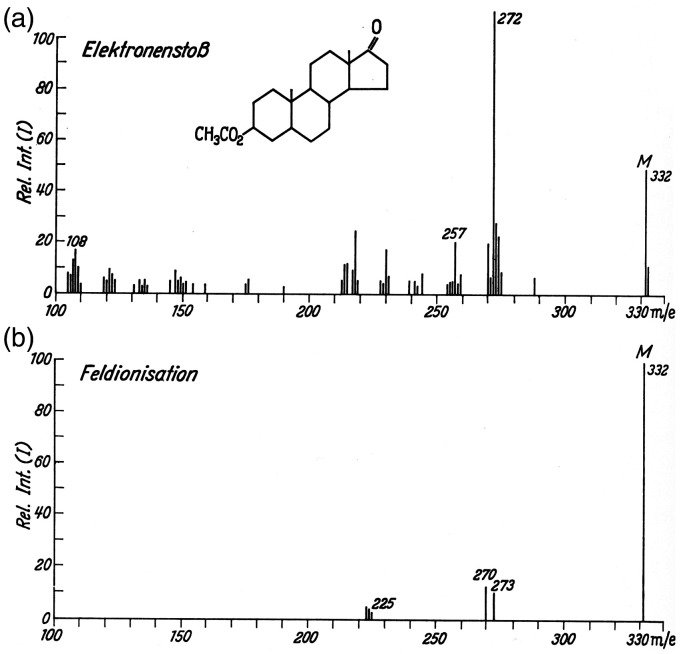
Mass spectra of 5α-androstan-3β-17-on-acetate as obtained by (a) 70 eV EI and (b) FI. In (b) the non-resolved pair of peaks at *m/z* 332 and 333 are represented as the sum of their intensities. Notes: *i*) To preserve the original character of the figure the peak labels have not been corrected to become M^+•^ and the abscissas are still labeled *m/e* rather than dimensionless *m/z*. *ii*) For a brief discussion of the spectrum cf. Example 2 in Ion Formation Processes in Field Ionization. Reproduced from Ref. [12] by kind permission. © Springer Nature, Heidelberg, 1965.

The story to be told here begins in 1953 with E. W. Müller’s observation of positive ion formation from a layer of metallic barium on a sharp tungsten tip in the strong electrostatic field of a field ion microscope.^1^ Analogous observations with other metals as well as with gases like H_2_, D_2_, O_2_, and C_2_H_6_ followed soon and the process was termed field ionization (FI).^1–5^ The pioneering experiments employed electric fields of up to 6 × 10^10^ V m^−1^ (6 V Å^−1^) formed at sharp etched tungsten tips. Ions emitted from there were admitted to a magnetic sector mass analyzer.^2,4,5^ In 1959, H. D. Beckey presented the first focusing field ionization ion source, which achieved a notable improvement by using electrostatic lenses to form a beam of the divergent ions emitted from the tungsten tip.^6^ The then chosen 2-mm distance between the tungsten tip (field anode) and a polished metal counter electrode with a small orifice (field cathode) has, by the way, become the standard until today. Within a few years, the optimized FI source was applied to analyze volatile liquids^7–11^ and then vaporizable solids.^12^ The solid samples were admitted by evaporation from a sample vial positioned next to the ionizing tip. Other metals such as gold and platinum were explored as tip materials, and next, the shape of the positive electrode was modified to sharp edges, e.g., razor blades,^13^ and 2.5-µm Pt wires that offered a 10^3^–10^4^ fold larger surface for ion emission^9–11,14^ In the early 1960s, the FI spectra of alkanes, amines, and some natural products impressively demonstrated the softness of FI as compared to electron ionization (EI, Figure 1).^10–12^

Since the mid-1960s, FI-MS faced competition from another quite soft ionization method, i.e., from chemical ionization (CI).^15–17^ By 1970, even high-resolution (HR) field ionization-mass spectrometry (FI-MS) had been accomplished^18,19^ and the developments were summarized in Beckey's first monograph.^20^

The next major advancement was the introduction of field desorption (FD), where analytes are deposited onto the field emitter (anode) from solution. In contrast to FI, there is no more need to evaporate the analyte before ionization, because the processes of ionization and immediate desorption of the incipient ions are occurring at the surface of the field emitter.^21,22^ Thus, FD-MS achieves an extraordinary softness of ionization, often delivering almost exclusively intact molecular ions.^23–26^ Moreover, FD is not limited to neutral molecular analytes. Instead it may as well be applied to ionic analytes, i.e., organic salts.^20,27–39^ The flourishing period of FD-MS began in the mid-1970s, which led to Beckey’s second monograph now covering FI-MS and FD-MS.^31^

From the mid-1980s, the strong competition from the easier-to-operate fast-atom bombardment (FAB)^40–42^ caused a decrease in applications of FD-MS. In the mid and late 1980s even more competition arrived by the advent of electrospray ionization (ESI)^43,44^ and matrix-assisted laser desorption/ionization (MALDI).^25,45,46^ The next – and until now – last monograph dealing with FI-MS and FD-MS by was published by L. Prókai in 1990.^47–49^ Nonetheless, FD-MS was never superseded, probably because it *i*) does not require any matrix and *ii*) essentially delivers molecular ion spectra.^38,50–54^

The most recent milestone was the development of liquid injection field desorption/ionization (LIFDI) in the early 2000s that allows for sample deposition under the exclusion of atmospheric oxygen and water.^55,56^ In addition to sampling under inert conditions, LIFDI also enables more robust and quicker operation than classical FI-MS and FD-MS procedures. Therewith, LIFDI expanded the range of FD applications and initiated a revitalization of the entire family of ionization methods now comprising FI, FD, and LIFDI. This reawakened a demand for FI, FD, and LIFDI at a time when magnetic sector instrument where phased out. Therefrom, a need arose to adapt these ion sources to current mass analyzers like orthogonal acceleration time-of-flight (oaTOF) and Fourier transform ion cyclotron resonance (FT-ICR) mass analyzers^57–62^ and Chap. 8 in Ref. [63].

**Figure 2.**
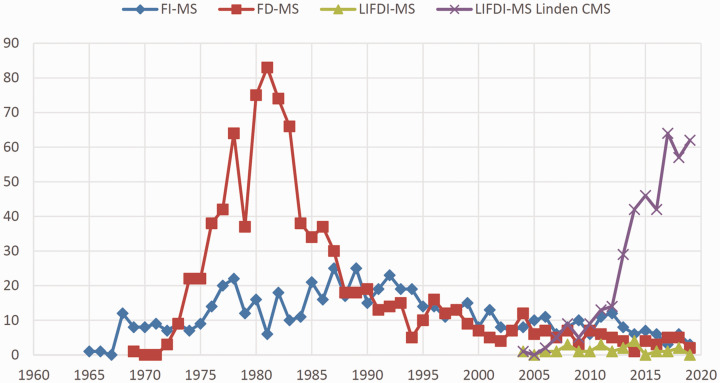
Number of annual publications as retrieved from Chemical Abstracts SciFinder based on searches for the terms “field ionization mass spectrometry”, “field desorption mass spectrometry” and “liquid injection field desorption ionization mass spectrometry” plus LIFDI-MS references collected from Linden CMS^64^ for the years 1959–2019. Note that the FI-MS data misses to reveal the pioneering publications on field ionization from 1953 onward until the term FI-MS has become used in the papers.

Searching Chemical Abstracts SciFinder for the terms “field ionization mass spectrometry”, “field desorption mass spectrometry” and “liquid injection field desorption ionization mass spectrometry” yields an estimate of the annual numbers of publications that have appeared on the respective topics, and thus, gives an estimate of the perceived relevance of these techniques. The last review showing such indicative literature data was published in 1989.^38^ The retrieved numbers, however, do not accurately reflect the number of published papers where any of these methods plays a role in some respect, as for example, entering the acronyms “FI”, “FD”, and “LIFDI” plus refining the answers by adding the key word “mass spectrometry” instead of the fully spelled terms results in somewhat different numbers. The results of these queries are combined in a graph (Figure 2) that illustrates the slow development of FI in the beginning, the impressive increase in research on FD and of uses of FD-MS as well as the decrease of interest in this technique when FAB appeared in the early 1980s.^40,41^ A second drop in FD-MS use can be observed in the mid and late 1980s caused by ESI^43,44^ and MALDI.^45,46^ Interestingly, the newer techniques had no notable effect on FI-MS, most probably as either of them is devoted to non-vaporizable samples, i.e., they were selectively harvesting from the realm of FD-MS. Based on the Chemical Abstracts SciFinder data, it appears that the introduction of LIFDI did not effect a major boost for this group of techniques. This is only true when looking at the SciFinder results that are highly dependent on the appearance of the search key word in the abstract of an article, whereas mere application or a brief mention in the experimental part do not effect a hit. Referring to the LIFDI manufacturer’s website^64^ providing a detailed list of all articles describing both developments and analytical applications of LIFDI-MS leads to a different impression. In fact, the advent of LIFDI has rejuvenated the entire family of ionization methods.

## Ion formation processes in field ionization

### Molecular ion formation

The process of field ionization differs markedly from that in electron ionization (EI) even though the product, a molecular ion, appears to be identical at first sight. However, in EI the molecular ions are formed with substantial excess energy causing their majority to undergo dissociation within the first microsecond of their lifetime:

$$\text{M}+{\text{e}}^{–}{\;}_{\left(\text{7}0\text{eV}\right)}\to {\text{M}}^{+˙}+{\text{2e}}^{–}$$

In FI, the electron is literally withdrawn without exciting the molecule. In essence, an internally supra-excited neutral loses an electron spontaneously without any need of energy transfer:

$$\text{M}\to {\text{M}}^{+˙}+{\text{e}}^{–}$$

**Figure 3.**
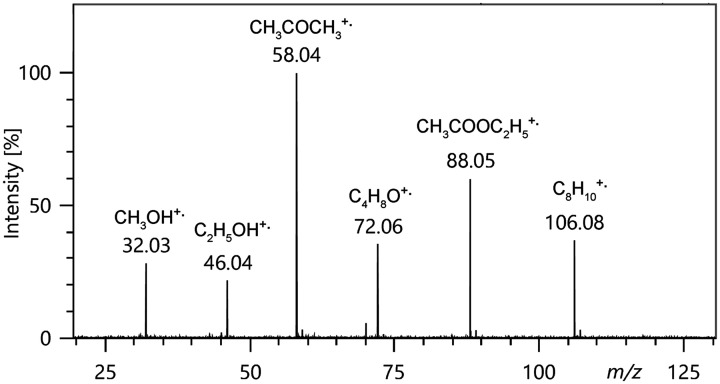
FI mass spectrum of a roughly equimolar solvent mixture composed of (*from left*) methanol, ethanol, acetone, tetrahydrofuran, acetic acid ethyl ester, and p-xylene showing the respective molecular ions. Fragment ions are essentially absent, thereby demonstrating the softness of field ionization.

**Figure 4.**
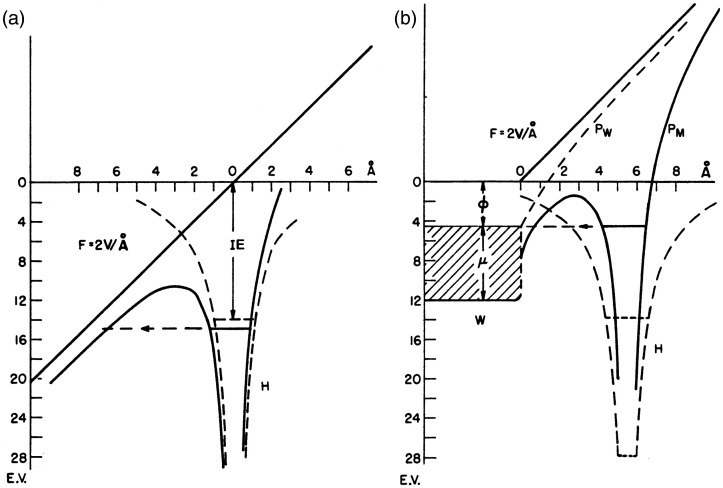
Potential energy diagram for an electron in a hydrogen atom in the presence of an applied electric field of 2 x 10^10^ V m^−1^ (2 V Å^−1^); (a) in free space, (b) near a metal surface. *IE*: ionization energy of the H atom, Ф: work function of the metal, µ: Fermi level. The dashed lines marked H are the hydrogen atom Coulomb potential in the absence of the field. Solid lines refer to potentials deformed by the external electric field. *P*_W_ is the sum of applied plus image potential, *P*_M_ is the atom potential. Adapted from Ref. [66] with kind permission. © Elsevier Science Publishers, 1994.

FI belongs to the category of auto-ionization processes^65^ and this is the reason for the extraordinary softness of FI (Figure 3). The ionizing effect of a strong electric field can readily be understood from the explication given by M. Inghram and R. Gomer for a single hydrogen atom.^2,5,66^ There are two cases to deal with, namely a hydrogen atom in the gas phase versus one adsorbed to a metal surface. In the presence of an electric field in the order of 2 V Å^−1^ the proton-electron potential of an isolated hydrogen atom gets heavily distorted in that the barrier for the electron to leave the nucleus in the direction towards the field anode becomes lowered by several electron volts (Figure 4). Thus, the electron can separate from the proton by tunneling towards the anode through the remaining potential barrier. The proton is then pushed into the opposite direction by means of the electric force and recombination is thus effectively avoided.

For an atom adsorbed to a metal surface, the proton-electron potential is also distorted towards the metal at positive polarity. Now, the electron may leave the proton by tunneling into the bulk metal through a potential barrier just several angstroms wide and a few electronvolts high.^31,47^ The adsorbed proton is then immediately driven away from the surface into the gas phase.

### Half-life of neutrals and ionization efficiency

As mentioned, in addition to high voltage, it requires sharp tips, edges, or thin wires to generate electric fields sufficient to effect field ionization. Sharper anode tips, i.e., smaller radii of the anode curvature, assist in building up electric fields strong enough to ionize even up to about 10 nm in the surroundings of the tip. It is of utmost importance to produce the strongest possible electric field, because the half-life of a neutral crossing that region needs to be in the range of several tens of picoseconds in order not to leave it still as a neutral.

The half-life τ of an atom or molecule can be calculated as a function of the finite probability for the electron to cross the lowered potential barrier. It is determined by the frequency ν with which the electron hits the barrier and by the quantum mechanical tunneling probability *D*^31^:

$$\tau =\frac{1}{\text{υ}D}$$

where D may be approximated by

$$D\approx \exp\left[–\frac{{0.68\;(IE\;–\;\Phi )}^{\frac{3}{2}}}{E}\right]$$

where *IE* denotes the ionization energy of the neutral, Ф the work function of the metal, *E* the electric field.^31,47^

Based on this relationship, the half-life for a hydrogen atom has been calculated to be in the order of 0.1 s at 0.5 V Å^−1^, of 0.16 ns at 1.0 V Å^−1^, and of just 17 fs at 2.0 V Å^−1 31^, i.e., 1.0 V Å^−1^ can be considered to suffice for effective ionization while passing a tip. The ion current, *I*_ion_, delivered by a field anode in a given volume element where field ionization can occur, can be calculated by:

$$I_{\mathrm{ion}}=e\;\frac{dn}{dt}\left[1-\exp\left(-\frac{t}{\tau }\right)\right]$$

where *e* represents the elementary charge, *dn*/*dt* the number of particles per time entering the volume, and *t* the residence time of that particle in this volume if there was no field ionization.^31,47^

### Doubly and triply charged ions by field ionization

FI may not only produce singly charged molecular ions. Doubly charged molecular ions, generally of lower abundance, can be formed when a second electron is abstracted before the M^+•^ ion leaves the area of ionizing field strength. This may occur via post-ionization of gaseous M^+•^ ions:

M^+•^ → M^2+^ + e^–^

In rare cases, even triply charged molecular ions can be formed if this happens twice^67,68^:

$${\text{M}}^{\text{2}}{\;}^{+}\to {\text{M}}^{\text{3}}{\;}^{+˙}+{\text{e}}^{–}$$

Apart from further gas phase ionization, an adsorbed ion, M^+•^_(ads)_, can be ionized for a second time before desorbing into the gas phase^69–71^:

$${\text{M}}^{+˙}{\;}_{\left(\text{ads}\right)}\to {\text{M}}^{\text{2}}{\;}^{+}+{\text{e}}^{–}$$

Thus, the field ionization process delivers M^+•^ ions, occasionally accompanied by M^2+^ ions and even by M^3+•^ ions of very low abundance.

### Formation of protonated molecules

The combination of molecules of low to medium polarity and low ionization energy with high electric field strength work in favor of molecular ion formation. Analytes of higher polarity or maybe some acidity due to exchangeable hydrogens as well as lower electric field strength and lower temperatures promote formation of protonated molecules, [M+H]^+^.^72^ The abundance of protonated molecules may even exceed that of molecular ions.

It has been demonstrated that [M+H]^+^ ions of acetone are formed via a field-induced proton-transfer reaction occurring in the physically adsorbed layer on the emitter surface.^73^ The mechanism of this field-induced reaction depends on the occurrence of tautomeric structures of acetone (or other neutral molecules). In addition to the [M+H]^+^ ions, [M–H]^•^ radicals are formed:

$${\text{M}}^{+˙}+\text{M}\to {\left[\text{M}+\text{H}\right]}^{+}+{\left[\text{M}–\text{H}\right]}^{˙}$$

The radical by-products formed upon field-induced hydrogen abstraction can initiate polymerization processes, and thus, cause high-mass product layers on the emitter surface.^73^

### FI mass spectra versus EI mass spectra

The early literature on FI often compared the new technique to the established EI mode to explore the capabilities of the new technique and to demonstrate the softness of FI.^12,74–76^ Some of these examples shall serve to illustrate the above mentioned ionization processes and the general appearance of FI mass spectra.

**Figure 5.**
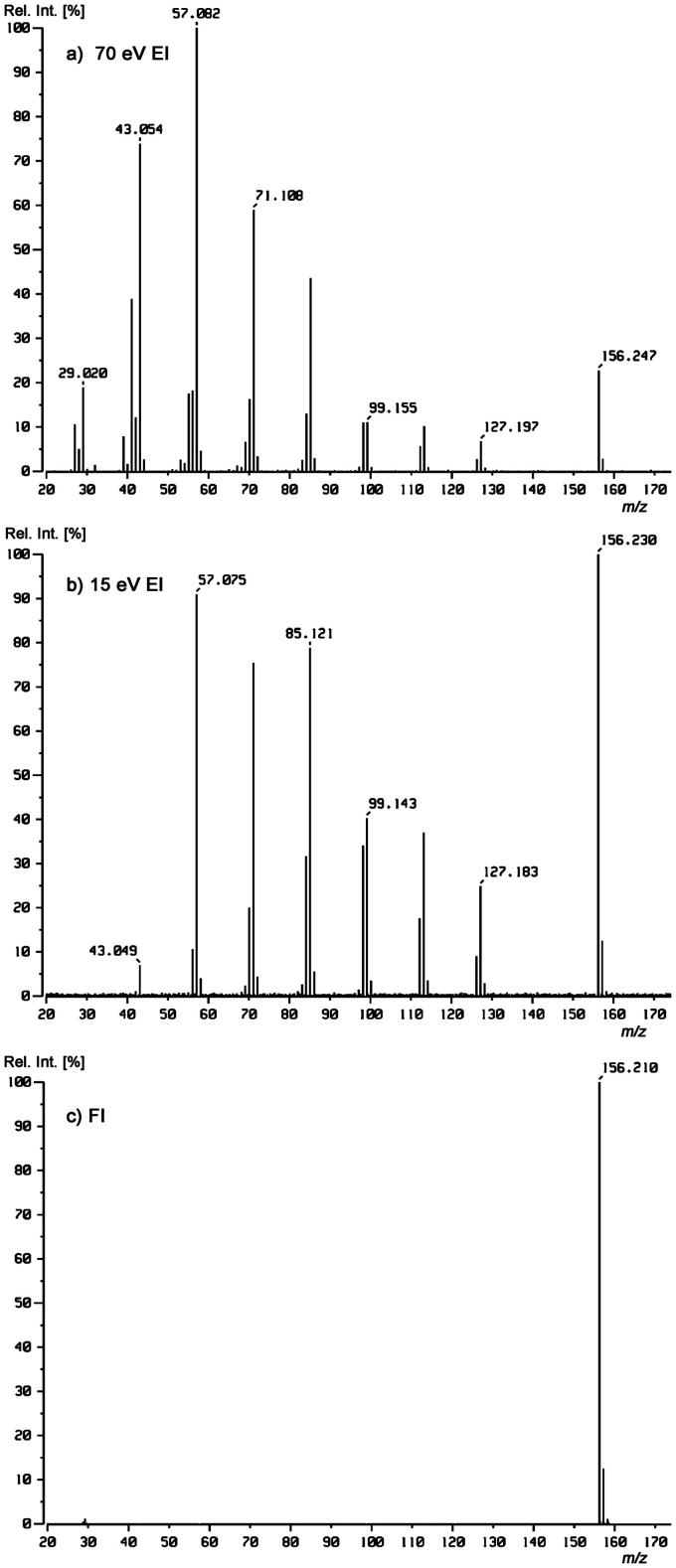
Comparison of (a) 70 eV, (b) 15 eV, and (c) FI mass spectra of *n*-undecane, C_11_H_24_, *M*_w_ = 156 u. For all three, the alkane was supplied via the reservoir inlet of a JEOL JMS-700 magnetic sector instrument. The FI mass spectrum was obtained using a 5 µm FI emitter at 10 mA emitter heating current.

**Example 1:** Classically, the relative intensity of molecular ions was increased by measuring EI mass spectra at low energy of the primary electrons, e.g., at 15 eV rather than at 70 eV.^77–79^ While the goal is achieved for the most part, the overall intensity of the spectra also drops notably due to decreased ionization efficiency at 15 eV. This is exemplified by comparing the respective spectra of *n*-undecane, C_11_H_24_, *M*_w_ = 156 u (Figure 5). In fact, the relative intensity of the molecular ion increases to become the base peak at 15 eV, just because less ions are able to decompose within 1 µs. However, the overall intensity of the signals is much lower and noise becomes visible at the baseline. In FI, apart from a tiny signal by C_2_H_5_^+^ at *m/z* 29, no fragments do occur as the internally cold molecular ions cannot decompose. The FI spectrum essentially delivers the M^+•^ ion and the correct isotopic pattern. Due to the lack of heteroatoms and in particular of acidic hydrogens, protonated molecules do not occur in this case.

**Example 2:** The 70 eV EI mass spectrum of 5α-androstan-3β-17-on-acetate shown in the beginning exhibits a number of fragment ion peaks along with the molecular ion peak at *m/z* 332 of about 50% relative intensity. In EI, the base peak at *m/z* 272 is due to an ion by loss of acetic acid, 60 u, from the M^+•^ ion. The FI mass spectrum obtained by evaporating the compound from a vial shows the molecular ion as the base peak while only a few fragment ion signals of low intensity do occur. In contrast to EI mode, acetyl loss, 59 u, yields an ion at *m/z* 273 via homolytic cleavage (Figure 1).^12^

**Figure 6.**
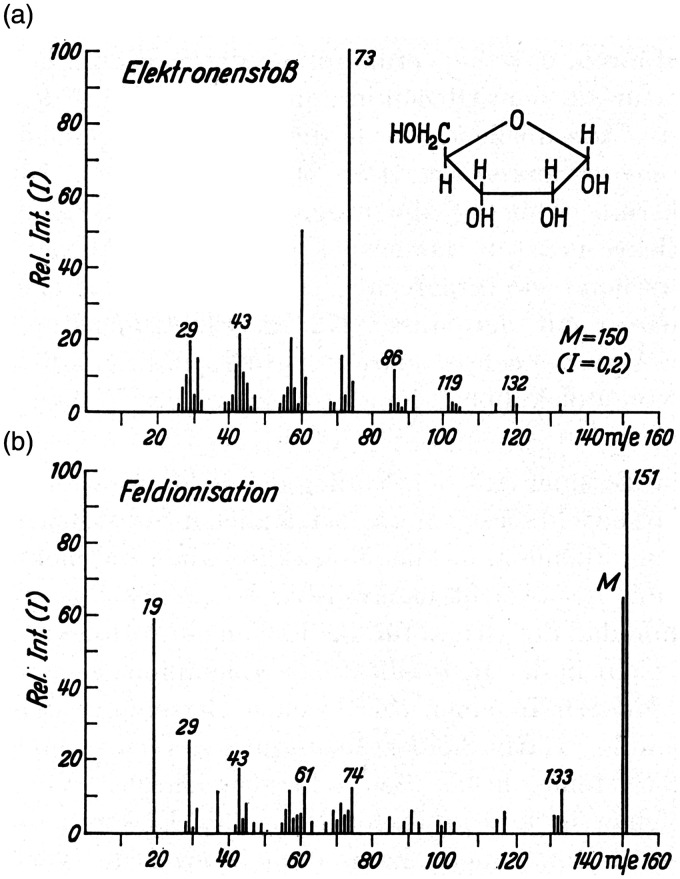
Mass spectra of D-Ribose as obtained by (a) 70 eV EI and (b) FI. This is an example showing the simultaneous formation of M^+•^ and [M+H]^+^ ions by FI. Note: To preserve the character of the original figure the peak labels have not been corrected to become M^+•^ and the abscissas are still labeled *m/e* rather than dimensionless *m/z*. Reproduced from Ref. [12] by kind permission. © Springer Nature, Heidelberg, 1965.

**Example 3:** The EI mass spectrum of D-Ribose shows the molecular ion at only 0.2% relative intensity and is dominated by a large number of intensive fragment ion signals. FI, in contrast, results in the simultaneous formation of M^+•^, *m/z* 150, and [M+H]^+^, *m/z* 151, ions as the base peak along with several fragment ion peaks (Figure 6).^12^ This corresponds to an about 1000-fold increase in ion current related to intact molecular species as compared to EI. While impressive, nowadays, FI would not anymore be tried to analyze carbohydrates as FD and other desorption/ionization techniques provide better results. Nonetheless, this presents a case where the formation of the protonated molecule is preferred over radical ion formation. Of course, the simultaneous occurrence of both molecular species is disadvantageous for the interpretation of the isotopic pattern.

**Figure 7.**
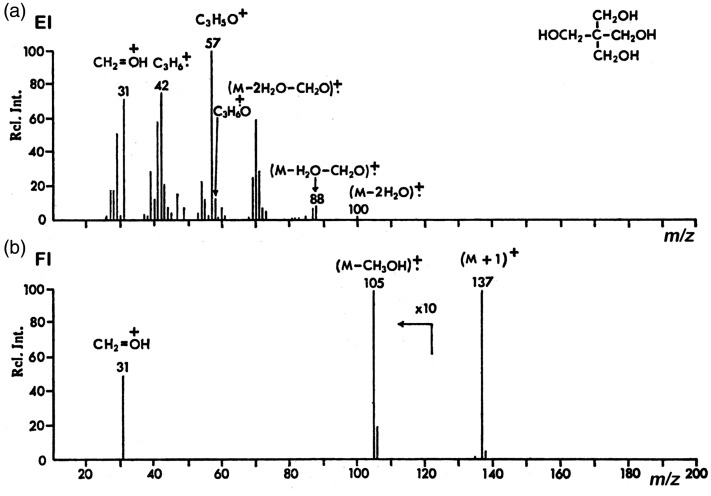
Comparison of (a) 70 eV EI and (b) FI mass spectra of pentaerythritol (2,2-bis(hydroxymethyl)propane-1,3-diol), C_5_H_12_O_4_. While in EI, the molecular ion is fully absent and the spectrum is dominated by fragments due to multiple water losses and homolytic cleavages, in FI mode, the protonated molecule, [M+H]^+^, *m/z* 137, is the main product accompanied by just two fragment ion peaks of low intensity (shown in 10x magnification). Reproduced from Ref. [74] with kind permission. © John Wiley and Sons, Chichester, 1972.

**Example 4:** Comparing the 70 eV EI and the FI mass spectra of pentaerythritol (2,2-bis(hydroxymethyl)propane-1,3-diol), C_5_H_12_O_4_, *M*_w_ = 136, shows the marked differences of these spectra. The EI spectrum shows no molecular ion peak but is dominated by fragments due to multiple water losses and several homolytic cleavages (Figure 7). The FI spectrum in contrast exhibits a signal due to the protonated molecule, [M+H]^+^, *m/z* 137, as the base peak that is accompanied by just two fragment ion peaks of low intensity, namely by CH_3_OH loss and an CH_2_OH^+^ oxonium ion, *m/z* 31, via α-cleavage of M^+•^.^74^ [M+H]^+^ ion formation is typical for FI of such highly polar compounds having several exchangeable hydrogens. In this case, the direct product of field ionization, the molecular ion, is even not observed.

## Ion sources for FI and FD

**Figure 8.**
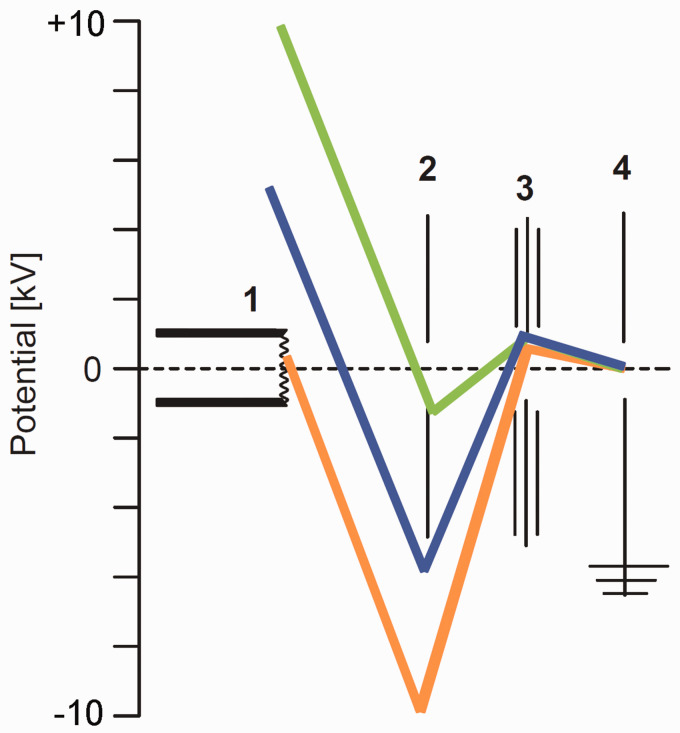
Potentials in FI and FD ion sources in correlation to the ion kinetic energy acceptance or requirement of the mass analyzer. The part numbers denote 1 field emitter, 2 counter electrode, 3 electrostatic lenses for ion beam focusing, 4 grounded entrance to mass analyzer. The ca. 10-kV potential difference between emitter (+) and counter electrode (–) can be realized by setting the emitter close to +10 kV (*green*), by keeping emitter and counter electrode at some intermediate level (*blue*) or by setting the emitter close to ground and the counter electrode to about –10 kV (*orange*). The ion kinetic energy at the analyzer entrance is thus always determined by the drop from emitter potential to ground.

As mentioned in the first section, in the early 1960s the shape of the field anode matured from sharp tungsten tips^4–6^ to edges of sharp blades^13^ and then to micrometer-thin wires^9–11^ as the latter combine small radii with a 10^3^–10^4^ fold larger surface advantageous for ion emission.^9–11^ Ever since, the basic shape of the field anode, generally termed field emitter or simply emitter has not changed, and thus, the emitter is represented this way in the general scheme of a FI/FD ion source (Figure 8; for details on the emitter see next section). To achieve the critical electric field at the emitter, a high voltage of about 10-kV needs to be applied across a 2-mm gap between emitter and counter electrode. This potential also defines the kinetic energy of the ions arriving at the counter electrode, which clearly exceeds the ion kinetic energy requirements of most mass analyzers. The workaround for this is to apply the potential equal to the analyzer’s requirement for ion kinetic energy to the emitter and to set the counter electrode to a negative voltage sufficient to establish the 10-kV difference. Ions having passed the counter electrode slit will thus decelerate towards the ion focusing electrostatic lens stack.

**Figure 9.**
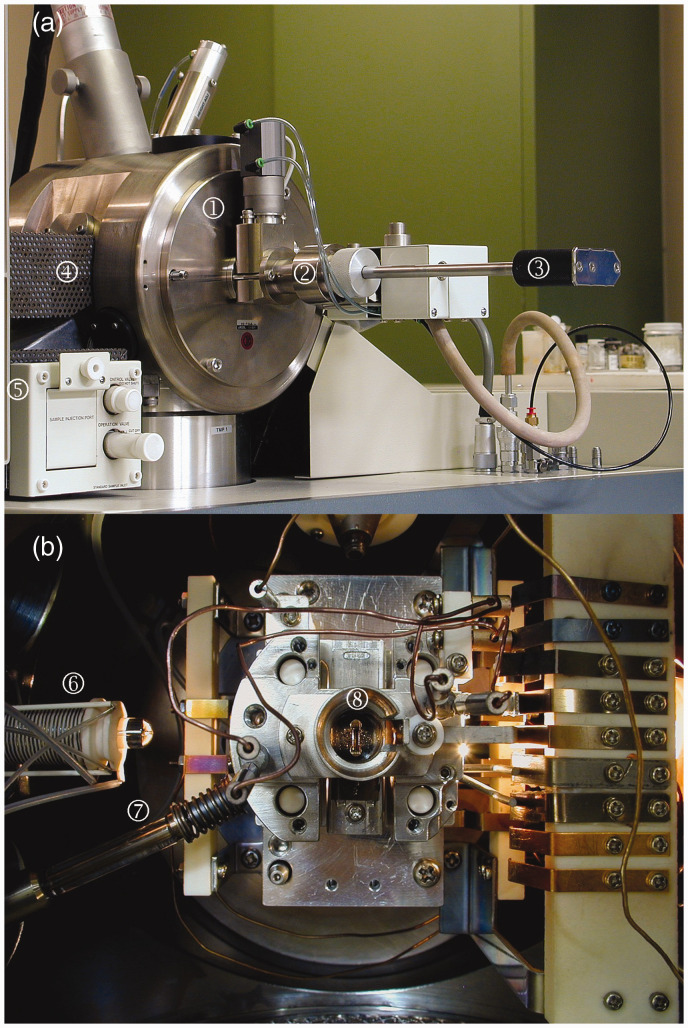
JEOL JMS-700 magnetic sector mass spectrometer (a) equipped with a FI/FD source; the numbers denote ① ion source housing, ② vacuum lock for FI/FD probe, ③ FI/FD probe, ④ GC interface, ⑤ reservoir inlet; the direct insertion probe would be located opposite to the GC interface. (b) The FI/FD source can be accessed via ⑥ GC interface, ⑦ reservoir inlet, and direct insertion probe (would be behind contacts on the right). Here, the ⑧ counter electrode is slotted for vertical emitter orientation, i.e., parallel to the analyzer entrance slit.

Gaseous or vaporizable samples may either be admitted via a reservoir inlet, via a gas chromatograph or by using a direct insertion probe as in EI or CI. This mode of operation is known as field ionization-mass spectrometry,^80^ i.e., is described by the same term as the ionization process. When the sample is deposited on the emitter surface to achieve desorption/ionization, this is termed field desorption-mass spectrometry.^21,22,31,47,81,82^ It can be inferred that the same ion source serves for both FI and FD operation as the modes are chosen just by the way of sample admission (Figure 9). While most FI/FD ion sources are optimized for these techniques, EI/CI/FI combination sources are also known for a long time.^83,84^ A glossary of FI/FD terms is compiled in Table 1.

**Table 1.** Glossary of FI, FD, and LIFDI.

Term	Note or acronym	Explication
Activated emitter		Emitter with thousands of sharp micro-needles covering the surface of the central wire. Opposed to a non-activated, i.e., bare wire emitter
Activation	*See* emitter activation	
Best anode temperature	BAT	Temperature of the field emitter resulting in (subjective) optimum appearance of a FD mass spectrum, i.e., good intensity, yet low level of fragmentation
Continuous-flow LIFDI	CF-LIFDI	Mode of LIFDI operation (cf. LIFDI) where a very low flow of analyte solution is continuously delivered to the emitter while high voltage is on
Counter electrode		Electrode opposed to the emitter serving as the second terminal to create the electric field at the emitter. The counter electrode, often a slotted plate of stainless steel, may also be realized by a pair of (round) rods
Emission-controlled emitter heating		Use of the actual emission current as a feedback to regulate the emitter heating rate as to stabilize the ion current at a preset level and avoid electric discharges due to too high ion currents
Emission control	*See* emission-controlled emitter heating	
Emitter		Electrode at positive potential (anode) with respect to the counter electrode (cathode) where the strong electric field is established to achieve field ionization of the analyte
Emitter activation		Procedure of growing micro-needles on the surface of an emitter wire to enhance the local electric field for largely improved ionization efficiency, typically performed by pyrolytic deposition of material on the wire in the presence of an electric field
Emitter heating current	EHC	Electric current (tens of mA) passed through the emitter wire for resistive heating. In FD to achieve mobilization of analyte layers on the emitter surface and generally for baking-off sample residues and to re-activate the emitter between runs
Emitter wire		Central wire of the emitter. A bare thin wire can serve as a non-activated emitter
FD probe		Dedicated direct insertion probe bearing the field emitter; needed for swapping of emitters and deposition of sample in FD operation. May also provide electric supplies to the emitter and counter electrode
Field anode	See emitter	Sink for the withdrawn electrons represented by the emitter in “normal” operation, i.e., positive-ion mode
Field cathode	*See* counter electrode	Represented by the counter electrode at negative potential in “normal” operation, i.e., positive-ion mode. Also serves to attract and accelerate ions towards the mass analyzer
Field desorption	FD	a) Process of positive ion formation of analyte molecules that are deposited on the emitter surface; in case of non-polar analytes the actual ionization may occur via field ionization. b) Experimental procedure to perform mass spectral analysis by field desorption
Field emitter	*See* emitter	Assembly providing a very sharp tip, edge or thin wire to be set to high positive potential with respect to a counter electrode in order to provide a region where a strong electric field can effect field ionization
Field ionization	FI	a) Process of positive ion formation by abstraction of the weakest bound electron(s) from a neutral atom or molecule by action of a very strong electric field. b) Experimental procedure to perform field ionization of gaseous analytes admitted to the emitter
LIFDI probe		Dedicated direct insertion probe bearing the field emitter and counter electrode. In addition to a FD probe, it is also equipped with a transfer capillary (cf. FD probe)
Liquid injection field desorption/ionization	LIFDI	Technique to admit sample, either as solution or sample vapor, to the emitter via a fused silica capillary while the emitter is in vacuum and positioned inside the ion source. The actual analysis is essentially performed by FD or FI, respectively
Negative-ion field desorption		Anions may desorb from the emitter, if potentials are reversed from standard operation, i.e., when the emitter is set to negative high voltage with respect to the counter electrode. Extremely rarely used mode
Sample transfer capillary	*See* transfer capillary	
Transfer capillary		Fused silica capillary fed through the rod of the LIFDI probe to admit sample vapor or sample solution from a septum vial to the emitter inside the ion source
Whisker		Term occasionally used for the microneedles grown by emitter activation
Wire emitter	See emitter	

### Sensitivity of field desorption

Ion currents in FI and FD are generally very low as compared to EI, often by orders of magnitude. The sensitivity of more recent magnetic sector instruments in FI is about 4 × 10 nA Pa^−1^ for the molecular ion of acetone, *m/z* 58, at *R* = 1000. This corresponds to an ion current of 0.4 × 10 pA at an ion source pressure of 10^−4^ Pa. In FD mode, the sensitivity is often stated for the [M+H]^+^ ion of cholesterol, *m/z* 387, at *R* = 1000. In case of a magnetic sector instrument (JEOL JMS-700), the sensitivity in FD is about 4 × 10^−11^ C µg^−1^, which is 10^4^ times less than the instrument achieves in EI mode and 10^3^ times less than in positive-ion CI mode. Fortunately, it is not all about ion currents. While the ion currents by FI and FD are by orders of magnitude smaller than those from EI or CI, the detection limits are not that bad. In general, about 0.1 ng of sample can yield a sufficient signal-to-noise ratio (S/N ≥ 10). The advantage of soft ionization is that most of the ion current corresponds to the molecular ion. There is also no chemical noise which leads to a very clean background only limited by the electronic noise of the instrument, thereby ensuring a good signal-to-background ratio. A comparison of 70-eV EI, positive- ion CI, negative-ion CI, and FI on average revealed a 200-fold lower total ion current from FI as compared to EI.^84^ However, the differences in molecular ion peak intensities were much smaller as FI spectra benefited from the concentration of the ion current on the molecular ions.

## Field emitters

**Figure 10.**
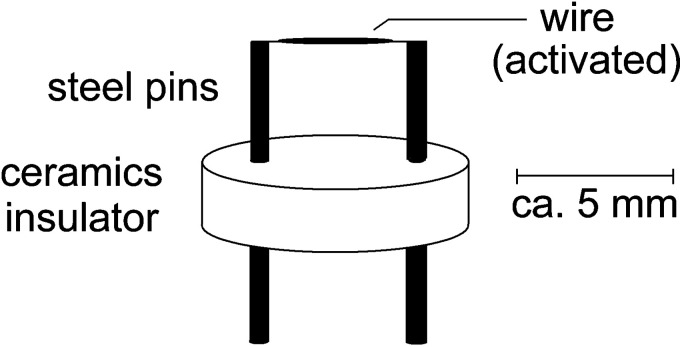
Schematic of a wire emitter. Two stainless steel pins are fixed in position by a ceramics insulator. The pins bear the emitter wire spot-welded to their upper ends, serve to deliver high voltage and emitter heating current to the emitter wire, and allow to plunge the emitter onto the tip of the probe. Reproduced with kind permission from Chap. 8 in Ref. [63]. © Springer International Publishing, Cham, 2017.

The field emitter presents the heart of FI and FD as its ability to generate a volume within the electric field is well above the critical level for field ionization to occur is key to effective analyte ion formation. As mentioned before, emitters are normally based on thin wires.^14,80^ Such a wire emitter assembly consists of two stainless steel pins that are fixed in position by a ceramics insulator. The pins bear the emitter wire that is spot-welded to their upper ends and allow to plunge the emitter onto the tip of the probe (Figure 10). In operation, the pins serve to deliver the high voltage and a heating current (tens of mA) to the emitter wire (cf. section Practical Considerations).

**Figure 11.**
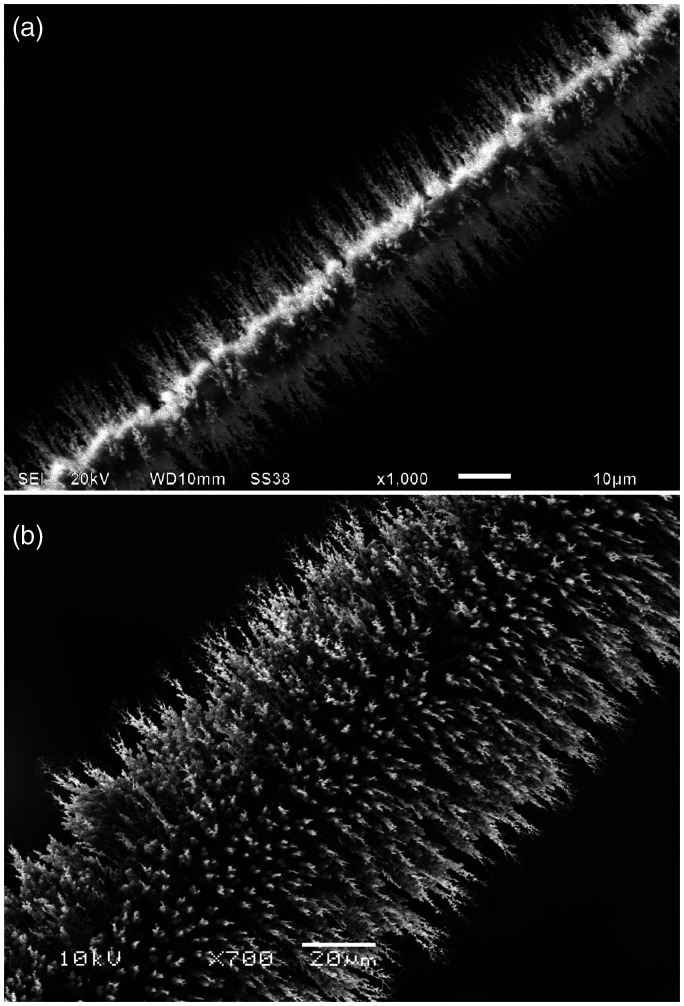
SEM micrographs of activated tungsten wire emitters. (a) FI emitter based on 5 µm wire bearing about 10 µm long needles rendering the central wire still visible. (b) FD/LIFDI emitter based on 13 µm wire with about 50 µm long needles. Courtesy Linden CMS, Leeste.

Bare wire emitters have in fact been used for quite some time.^14,85–87^ However, the electric field strength at the emitter surface can substantially be increased when the wire is covered with a large number of dendritic microneedles (aka whiskers, Figure 11).^80^ The process of growing such microneedles on the wires is known as emitter activation. Today, activated emitters are all based on tungsten wire where needles have been grown by so-called high-temperature activation, i.e., by vacuum pyrolysis of carbon-rich organic compounds on the hot wire in the presence of an electric field to direct needle growth.^88–90^ While the original procedure for high-temperature activation of 10-µm tungsten wires with benzonitrile vapor takes several hours,^88^ it may be speeded up by reversal of the polarity of the high voltage during activation^89^ or by employing indane or indene.^90^ The indene-based activation serves for the production of commercially available emitters.

These emitters are very fragile, because the high-temperature activation causes carbon atoms to diffuse into the tungsten wire to form brittle tungsten carbide. Therefore, activated emitters rather exhibit the mechanical properties of a ceramics material than those of an elastic metal wire.

Rarely used but certainly worth mentioning are alternative techniques to prepare activated emitters, e.g., microneedles can be grown by decomposing hexacarbonyltungsten, W(CO)_6_, on a cathode during an electric discharge.^91^ Emitters were also prepared from silicon,^87^ cobalt,^92^ nickel,^93^ MoCr alloy,^94^ or silver.^95^ Even fractured graphite rods have been proposed as emitters.^96^ The articles cited in this section are also recommended because of the unique SEM images of the activated emitters and whiskers.

The SEM micrographs in Figure 11 show two types of emitters, i.e., a dedicated FI and a FD/LIFDI emitter. The emitter optimized for FI is based on a 5 µm central tungsten wire and is bearing about 10 µm long needles. The FD/LIFDI emitter is made from 13 µm wire and has about 50 µm long needles. The fewer and shorter needles of the FI emitter are assumed to cause less mutual shielding of the electric field, which overall results in higher effective field strength. The shorter needles of the FI emitter also help to cool down faster after flash-heating (cf. Gas Chromatography-Field Ionization). The longer and tighter “furr” on the FD emitter is better suited to accommodate more sample in thin layers on its surface.

## Ion formation in field desorption

### Field ionization in FD mode

**Figure 12.**
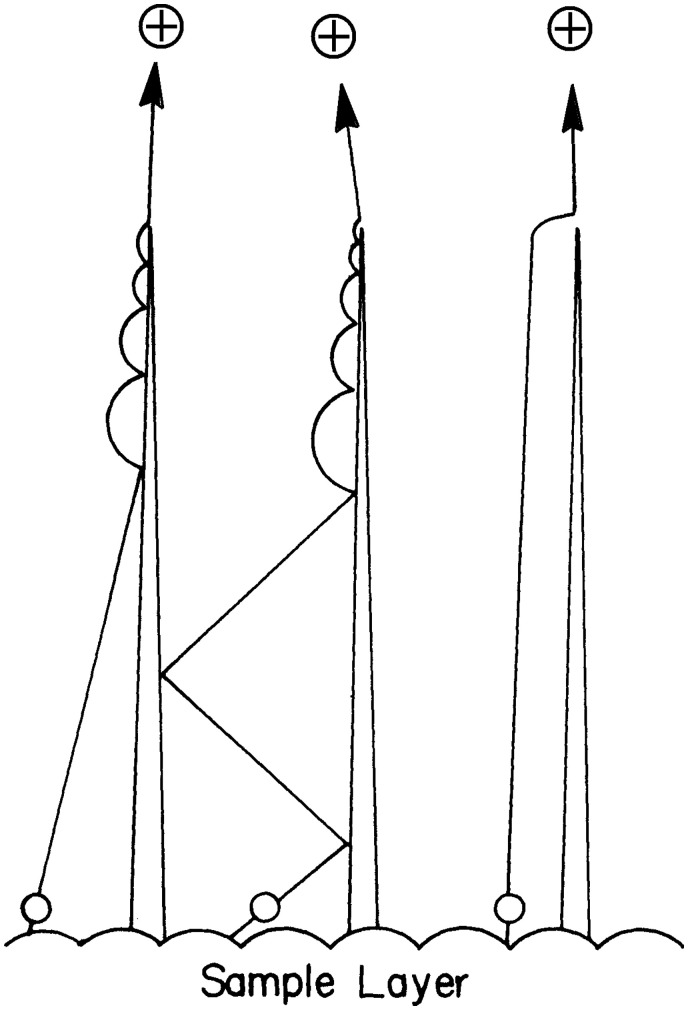
Transport of polarized analyte molecules from the bottom of the emitter wire along the shanks of the microneedles towards the tips where strong electric field can effect FI. Reproduced from Ref. [94] by kind permission. © Elsevier Science Publishers, 1981.

Field ionization as a process also represents a major mechanism of ion generation of nonpolar compounds in field desorption from activated emitters.^28,29,38^ Assuming that the analyte molecules are deposited in thin layers on the shanks of the microneedles or at the “bottom” between them, it will be required that the molecules can reach the locations of ionizing electric field strength at the tips of the whiskers. Analytes of low polarity are first polarized by action of the electric field, and consequently, experience the electric force causing them to move along the field gradient. This obviously requires some mobility of the polarized molecules. To achieve this the emitter is generally heated by passing a low current through it, the so-called emitter heating current (EHC). Molecules thus become mobile within the layer as the emitter temperature rises and may finally approach the needle tips to become field ionized. The transport may either proceed via the gas phase by “jumping” from adsorption site to adsorption side or by surface diffusion in the melting layer (Figure 12).^94^ The relative importance of field ionization as the pathway of analyte ion formation in FD decreases along with increasing polarity of the analyte. In certain cases such as sucrose, for example, it is not easy to decide whether gas phase mobility of the neutral and molecular ions jointly formed by FI still play a role^97,98^ or not.^99^

### Desorption of preformed ions

Field ionization does not anymore play a role for ion formation of very polar compounds where protonation or cationization prevail.^81^ Then, the electric field is only needed to effect desorption of preformed ions from the surface into the gas phase. The field strength required for desorption of [M+H]^+^ or [M+alkali]^+^ ions existent in the condensed phase is lower than that for field ionization or for field-induced [M+H]^+^ ion formation.^86,100–103^

The lower requirements for electric field strength are demonstrated by FD from bare wire emitters, because FD mass spectra of tartaric acid, arginine, pentobarbital, and other compounds were obtained when alkali metal salts were added to the organic compounds.^81,100^ The FD mass spectrum of arginine, for example, exhibited [M+H]^+^ ions, *m/z* 175, as well as [M+Na]^+^, *m/z* 197, and [M+K]^+^, *m/z* 213, ions due cationization by alkali ions. In addition, [2M+H]^+^, *m/z* 349, cluster ions were observed.^86^

**Figure 13.**
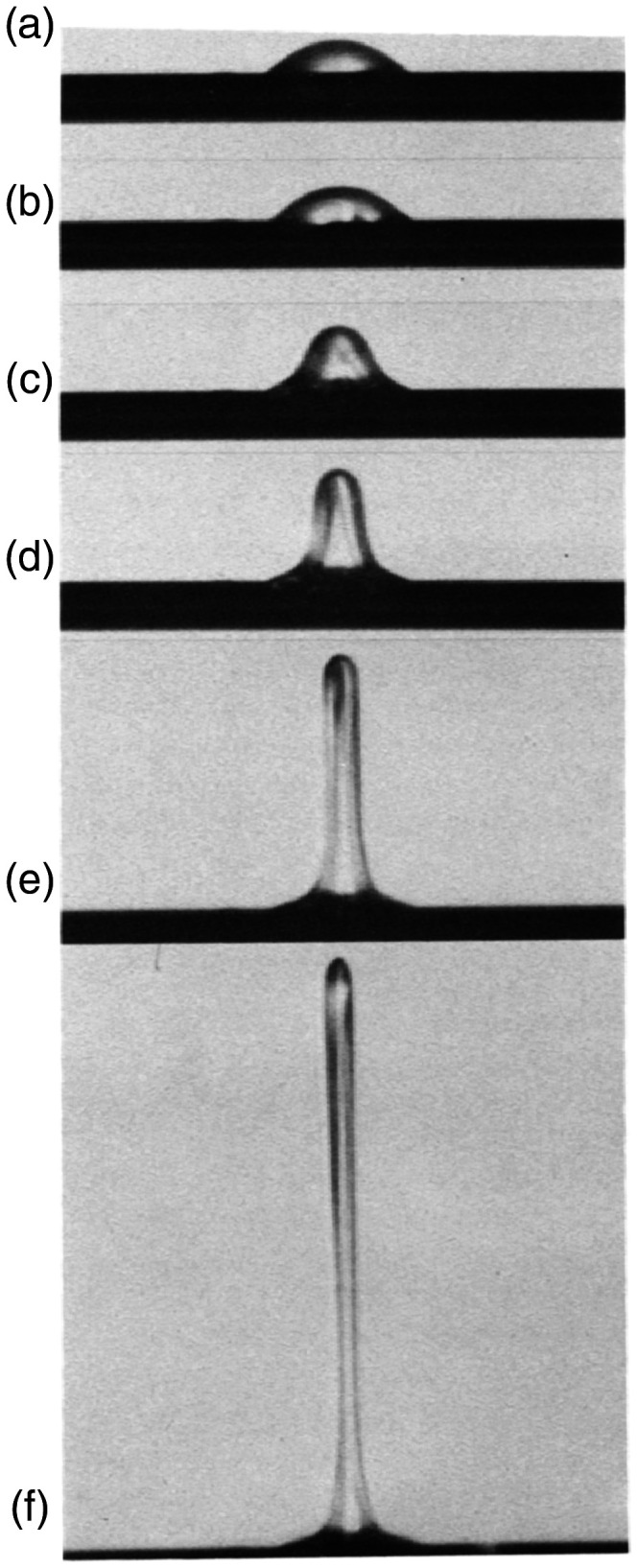
Protrusion developing from a concentrated aqueous solution of sucrose and NaCl applied to a bare wire emitter as observed via a microscope attached to a FD source. The onset of [M+Na]^+^ ion desorption was observed at point (f). Adapted from Ref. [106] by kind permission. © Elsevier Science Publishers, 1984.

**Figure 14.**
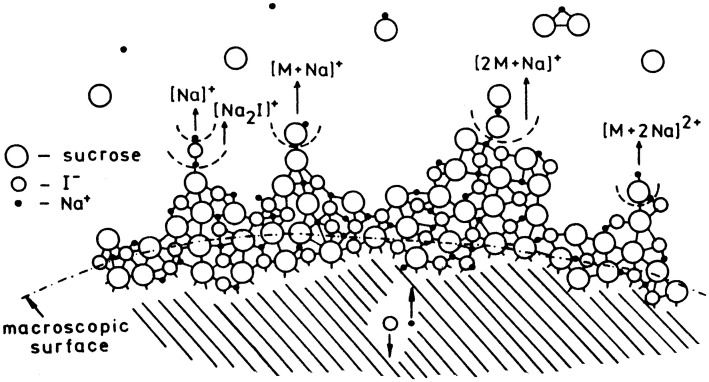
Representation of the extraction and desolvation mechanism for various ions from a nearly solid or glassy state of a mixture of sucrose and NaI. On a molecular scale, the surface is rough, almost free of solvent, and of low ionic conductivity. Due to some mobility in the layer, charge separation leads to protuberances, desolvation of ionic species, and finally their release into the gas phase. A continuous reconstruction of the surface provide continuous supply. Reproduced from Ref. [106] by kind permission. © Elsevier Science Publishers, 1984.

The question remains how ions are leaving the surface on a molecular scale. Two somewhat competing models were suggested, first field-induced desolvation^104–106^ and second ion evaporation.^107,108^ Both models assume that ions are already formed in the condensed phase and are subsequently desorbed into the gas phase by action of the electric field. The first step should be charge separation within the adsorbed layer. The model of F. W. Röllgen was developed from the microscopic observation of protuberances from glassy sample layers (Figure 13).^104,105^ These protuberances have a field-enhancing effect that allows ions to escape into the gas phase (Figure 14).

The model of P. J. Derrick assumes microscopic protuberances of about thousand-fold lower size.^107^ This model emphasizes the role of mobility on the molecular scale in contrast to microscopic viscous flow of the surface layer. Solvated ions are supposed to be drawn towards the surface, which then gets deformed to develop a protrusion. As this protrusion is expanding the local electric field is enhanced, thereby causing further elongation and reduction of the radius at the tip until ions leave the solvate behind and evaporate from there.

### Negative-ion FD

The first report on the use of FD in negative-ion mode describes the detection of small anions like OH^–^, F^–^, Cl^–^, NO_3_^–^, Br^–^, BF_4_^–^, and HSO_4_^–^ after application of inorganic salts to a tungsten surface.^109^ Five years later some organic anions like sulfonates, carboxylates and others were analyzed this way.^110–114^ Thus, negative-ion FD-MS can, in principle, be used for the direct detection of the anion A^–^ of a salt CA and of cluster ions of the general composition [C_n–1_A_n_]^–^ .^111,115^

However, there is no inverse FD process that would represent an electron transfer from the emitter to the analyte to yield the electron capture product M^–•^. From activated emitters, electrons are emitted below the threshold for negative ion formation. The resulting electron emission current causes a spark discharge that leads to the destruction of the emitter. Blank wire emitters and low emitter voltages may avoid such problems.^112^ Under these conditions, neutral analytes can form [M–H]^–^ ions or adducts with anions like [M+Cl]^–^ ions.^116^ Essentially, negative-ion FD-MS has remained an exception^115–120^ and shall not be pursued any further in this Account.

### Emitter heating versus analyte decomposition

From the above description of the various processes leading to formation of ions and their deliberation into the gas phase, it follows that some mobility within the sample layer is required. While this prerequisite is fulfilled in case of viscous liquids and of analytes of waxy consistency, crystalline layers are going to prevent molecules from moving towards the tips of the whiskers. It is therefore common practice to heat the emitter wire by passing a current through it. The emitter heating current (EHC) is generally ramped during the spectral acquisition and analyte ion formation starts when the temperature is sufficient to provide diffusion of the molecules to the whisker tips. The onset of analyte ionization and desorption depends on its intrinsic properties as well as on the extraction voltage and the actual emitter in use. The temperature of the emitter may need to reach several hundred degrees Celsius.^121,122^ It is not straightforward to correlate the applied EHC with the actual emitter temperature as this strongly depends on physical parameters like diameter of the central emitter wire, length of the activated zone, and length of the microneedles grown on the emitter. Generally, the EHCs for activated 10 µm wires are in the range of 0–50 mA, for 13 µm wires in the range of 0–80 mA. At slightly higher values the emitters start to glow (800–1000°C).^123,124^ Moderate glowing is used to bake off residual sample after the acquisition and to reactivate the emitter before the next run.

Consequently, there is some risk of thermal decomposition of the analyte in FD-MS. Generally, desorption precedes the thermal decomposition of the analyte but increasing emitter temperature can effect fragmentation. In practice, a balance is required between a temperature to obtain spectra of good signal-to-noise ratio while the level of fragmentation is still low or moderate at least. This condition is achieved at the so-called best anode temperature (BAT), of course a highly subjective value.^31,47^ The BAT has nonetheless been discussed quite frequently in the context of optimized FD spectral acquisition.^125–132^

Alternatively to acquiring FD spectra at a constant EHC ramp, the acquisition can be performed in a emission-controlled manner to avoid emitter rupture due to electric sparking.^133–135^ With emission-controlled EHC, the EHC ramp is flattened or paused when the ion current approaches a preset limit, e.g., 1 µA, and resumed when the emission decreases.

As a result of EHC ramping, the total ion chromatogram (TIC) of a FD analysis normally shows a section of very low intensity up to the onset of desorption. From there, the TIC often rises steeply for several tens of seconds and finally drops upon consumption of the sample (cf. Applications of FD-MS).

### Summary of ion formation by FI and FD

One may now summarize the various types of ions formed in FI-MS and FD-MS based on the various processes of odd-electron and even-electron ion formation in FI and FD. What ions are preferentially formed also depends on the interplay of physical and chemical properties of the analyte and on emitter properties and temperature (Table 2)^47^ and Chap. 8 in Ref. [63]. As FI requires supply of sample vapor, molecular analytes, M, of low to medium polarity are suited best, whereas salts, CA, can only be dealt with in case of unusual thermal stability.^136^ FD can essentially deal with all classes of analytes, and in fact, FD handles ionic compounds very easily. At higher partial pressure of the sample in the FI source or at higher sample load on the FD emitter, respectively, cluster ions can also be formed. As a desorption/ionization technique, FD is much more prone to cluster ion formation than FI. Cluster ions of salts, [C_n_+A_n–1_]^+^, are spaced at Δ(*m/z*) = *M*_CA_, thus allowing to identify the anion by subtracting the mass of C^+^ from that of Δ(*m/z*) corresponding to the mass of CA.

**Table 2.** Ions formed by FI and FD.

Analyte	Ions formed in FI	Ions formed in FD
Nonpolar, M	M^+•^, occasionally low abundance of M^2+^, rarely M^3+•^ or [M+H]^+^	M^+•^, occasionally low abundance of M^2+^, rarely M^3+•^ or [M+H]^+^
Medium polar, M	M^+•^ and/or [M+H]^+^, occasionally low abundance of M^2+^, at higher sample pressure [2M]^+•^, [2M+H]^+^	M^+•^ and/or [M+H]^+^, [M+alkali]^+^, occasionally M^2+^, rarely M^3+•^, at higher sample load also [2M]^+•^, [2M+H]^+^, [2M+alkali]^+^
Highly polar, M	[M+H]^+^, [2M+H]^+^	[M+H]^+^, [M+alkali]^+^, [2M+H]^+^, [2M+alkali]^+^, higher cluster ions possible
Ionic, [C+A]	Decomposition. In rare cases C^+^, CA^+•^, [2C+A]^+^	C^+^, [2C+A]^+^, occasionally [3C+2A]^+^, higher cluster ions possible, rarely CA^+•^

## Liquid injection field desorption/ionization

**Figure 15.**
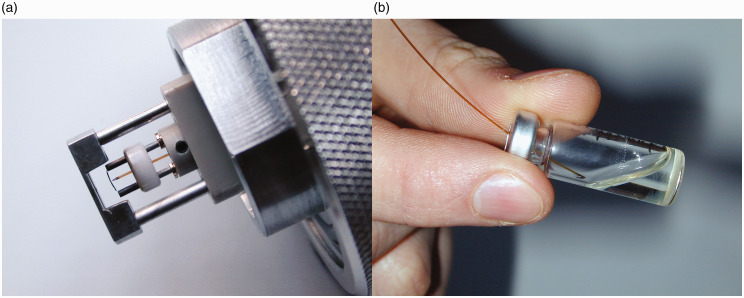
LIFDI probe setup and operation. (a) The LIFDI emitter has a feedthrough for the sample transfer capillary to be pushed close from the backside. The setup shown here also carries the counter electrode at the tip of the probe. (b) Supply of sample to the emitter by dipping the atmospheric end of the transfer capillary into the sample solution stored in a capped septum vial. Adapted from Ref. [56] with kind permission. © SAGE Publishing, 2004.

The invention of liquid injection field desorption ionization (LIFDI) has expanded the application of FD-MS to reactive analytes that otherwise would undergo immediate decomposition by reacting with atmospheric oxygen and/or water during conventional emitter loading.^56,137^ This is achieved as the analyte solution can be handled under inert conditions rather than loading a drop to the emitter at the open atmosphere. In LIFIDI, the analyte solution is transferred from a capped septum vial through a fused silica capillary to the emitter that is already inside the ion source vacuum. Sample transport is accomplished by the ion source vacuum that is sucking in the solution as long the atmospheric pressure end of the transfer capillary is dipped into the sample solution. Careful alignment of the sample transfer capillary with respect to the emitter axis provided, a small volume of solution is dissipated on the emitter. Thereafter, the solvent evaporates within seconds. As the transfer capillary approaches the emitter from the side opposite to the counter electrode, it does not interfere with the emission of ions towards the mass analyzer. Thus, there is no need to remove the capillary during the measurement or to change the positioning of the emitter inside the ion source (Figure 15). When the high voltage is switched on, the emitter slightly bends toward the counter electrode and further reduces the risk of getting into contact with the tip of the transfer capillary.

It turns out that LIFDI also simplifies the procedure of emitter loading as it circumvents the manual procedure (cf. Practical Considerations) and enables multiple sample runs without breaking the vacuum between successive measurements. This also reduces the need for frequent tuning of the ion source potentials.^56,138,139^ The sample load is generally reduced, because LIFDI requires more dilute sample solutions and delivers smaller drop sizes to the emitter than conventional emitter loading. Moreover, this enables faster ramping of the EHC, e.g., at 20–50 mA min^−1^. Taking these advantages together results in a remarkable reduction of measurement times in LIFDI as compared to conventional FD-MS and in a reduced risk of emitter damage.

By adjusting the transfer capillary entrance in the vapor phase over a volatile analyte it also allows for the acquisition of FI spectra. This is particularly useful for instrument tuning based on the FI signals of toluene or acetone, for example. Apart from these practical aspects, LIFDI delivers FI and FD spectra equivalent to those obtained by the classical setups. Thus, with the exception of insoluble samples that might require a suspension to be loaded onto the emitter, there is no reason not to run a FD-MS experiment by using the LIFDI equipment. LIFDI even allowed for the construction of an automated sampling system.^140–142^

## Practical considerations

**Figure 16.**
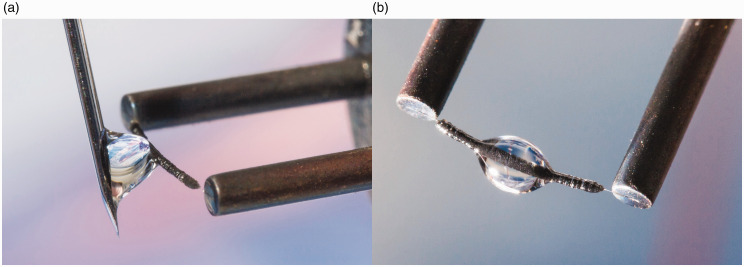
Manual emitter loading by using a 10 µl microliter syringe. (a) A drop of 1–2 µl at the tip of the needle is attached sideways to the activated emitter while strictly avoiding any other contact than to the liquid surface. (b) Gently retracting the needle by a movement to the side and up leaves the drop hanging like dew on a spider’s web. One can see that the liquid spreads out almost to the ends of the activated zone. After evaporation of the solvent the emitter is ready for insertion into the ion source.

Doing FI, FD, and LIFDI analyses can be joyful or frustrating, depending on how one handles the emitter and how one performs the actual experiment. As activated wire emitters are extremely fragile, the wire will inevitably break upon the slightest touch, e.g., plugging an emitter at the probe tip needs to be done the right way. Transferring sample solutions with a syringe needle requires the syringe to be operated in a way that the contact will only occur between the liquid and the emitter surface whereas contact with the needle must be avoided (Figure 16). There is also some risk of emitter rupture in operation by electric discharges. Apart from destruction, either accidental or by electric discharge in operation, the actual number of samples an emitter can be used for has no fixed limit. Sample residues that cannot be baked off like metal oxides formed upon decomposition of metal complexes as well as sintering due to baking at the upper EHC limit cause degradation of the emitter. An extreme case occurred in the author’s laboratory when a single run with C_119_ fullerene caused almost complete loss of emitter activity. Presumably, at the very high EHC required to generate C_119_^+•^ ions, decomposition competed with C_119_^+•^ ion formation, thereby causing the carbon to cover the fine needle tips. Adequate handling provided and depending on the actual mode of operation and type of samples, emitters normally last for 10 to 30 measurements.

### Emitter handling

To mount a fresh emitter at the probe tip, follow this procedure: Firmly grasp the new emitter in the package by using round-tipped metal tweezers. To do so, align tweezers in parallel with the emitter wire in a way that both steel pins of the emitter are simultaneously grasped before drawing it out vertically. The pins are much wider than the activated emitter and ensure sufficient distance to the delicate part. Then plug the emitter at the tip of the probe by pushing the lower part of the pins into the sockets. In case of using a LIFDI probe also carefully align the transfer capillary with the central hole of the ceramics before pushing the emitter into the sockets.

Prior to using the emitter for the first time, transfer it into the ion source, allow some time for the vacuum to recover, gradually apply high voltage, and then bake the emitter twice for about 1 s by passing through an EHC close to the respective limit (ca. 25–30 mA for FI emitters, ca. 50–60 mA for 10 µm FD and 80–90 mA for 13 µm FD/LIFDI emitters). Now it is ready for ion source tuning and analytical use. Then, the recommended procedure to run an analysis depends on whether this is intended to be performed in FI, FD or LIFDI mode. For any of these techniques preferably keep the ion source at low temperature (40–80°C).^143^

### FI Analyses

For highest signal intensity tune all relevant ion source voltages after the emitter is in its final position and at exactly the potential to be used in operation, i.e., 10 kV relative to the counter electrode in most instances. Bake the emitter once. Now apply no or very low EHC (0–10 mA) to the emitter. The gaseous analyte may then be admitted via the reservoir inlet, a gas chromatograph (special rules apply, cf. GC-FI) or from a crucible on a direct insertion probe (DIP). In case of using a DIP, it needs to be inserted before switching on the emitter high voltage. After completion of the acquisition interrupt sample introduction and switch off the high voltage. Remove the DIP if it has been used. Bake the emitter before starting the next run.

### FD analyses

Prepare a dilute solution of the analyte (0.1–2.0 mg ml^−1^) in a volatile organic solvent. Insoluble compounds may be finely suspended using an ultrasonic bath. Use a small microliter syringe and apply a drop of the solution as illustrated in Figure 16.^94^ Dedicated micromanipulators may be used for syringe handling,^22,80^ but with steady hands and some exercise it can well be accomplished manually. Do not load large drops as they tend to fall off the wire, or even worse, excessive analyte solution may spread along the steel pins thereby causing cross-contamination in the subsequent runs. Rather repeat sample application two or three times if the amount in a single drop turns out too low for acquiring a good spectrum. Allow for complete evaporation of the solvent before inserting the probe into the vacuum lock and switch on the high voltage only after the high vacuum has fully recovered. Start an EHC ramp in accordance with the speed of acquisition achieved by the mass analyzer in use (magnetic sector 4–8 mA min^−1^, time-of-flight 20–50 mA min^−1^). After completion of the measurement bake the emitter to remove sample residues and switch off the high voltage.

### LIFDI analyses

**Figure 17.**
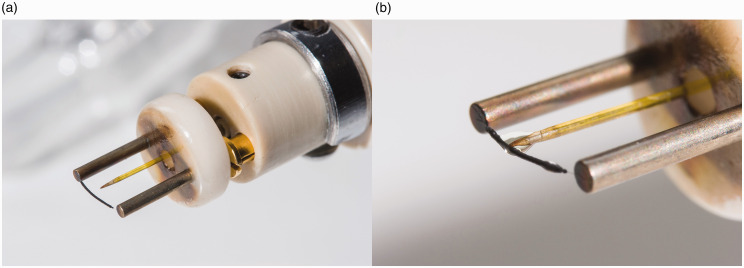
(a) Emitter for LIFDI with a central hole in the ceramics part to feed through the transfer capillary. The capillary needs to be adjusted with respect to the emitter as to enable smooth transfer of sample solution onto the emitter. (b) Wetting of the emitter with sample solution with the tapered tip adjusted close to the emitter surface but still just not touching it. The liquid is spreading along the activated zone. In normal LIFDI operation, this step would be performed inside the ion source.

**Figure 18.**
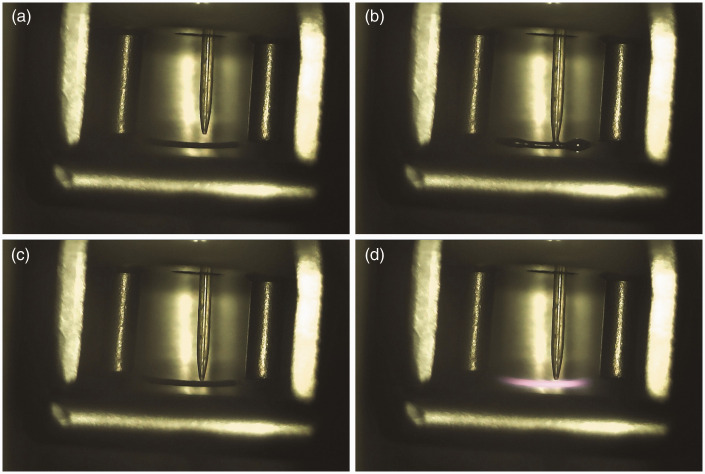
Emitter observation in LIFDI-MS; here with a probe carrying also the counter electrode. Views are screenshots from a USB microscope camera. (a) After insertion of the probe, the tapered tip of the transfer capillary is still not close to the emitter wire. (b) The capillary has been moved in as to almost touch the needles of the activated emitter. The gap is merely visible. (c) Sample solution is flowing onto the emitter. (d) Emitter baking.

With the LIFDI emitter in place at the probe tip adjust the sample transfer capillary. First move the capillary inward until the tapered tip comes close (0.5–1.0 mm) to the emitter wire (Figure 17). Next, align the tip with the emitter while observing it along the capillary axis through a 10× loupe. After insertion of the probe into the ion source, the capillary needs to be moved until it almost touches the emitter (10–30 µm) as to enable the liquid to bridge the gap and flow onto the emitter. A test with neat solvent will show whether this has been accomplished. The latter two steps rely on the microscope camera for emitter observation inside the ion source (Figure 18). After baking the emitter and ion source tuning using solvent vapor admitted via the capillary (cf. FI) the setup is ready for deposition of a sample. Sample solutions should be more dilute than in FD (0.1–0.2 mg ml^−1^) to avoid blocking of the capillary. Different from FD, suspensions are not acceptable in LIFDI mode. Most organic solvents can be used. Toluene, tetrahydrofuran, acetone, acetic acid ethyl ester, and methanol work best, dichloromethane, chloroform, and diethylether are acceptable, acetonitrile or benzene will freeze in vacuum thereby disrupting the sample transfer. Then proceed as in FD mode until the acquisition is completed. The transfer capillary should be flushed with solvent and the emitter be baked before commencing the next run.

## Mass analyzers for FI, FD, and LIFDI

At the time of the development of FI and FD, magnetic sectors essentially were the only mass analyzers available. Publications on FI and FD from the 1960s to the late 1990 were governed by these analyzers and the most prominent ones to recall are (sorted by company and in chronological order) the MAT CH5, MAT 311A, Finnigan MAT 90, Finnigan MAT 95, and Finnigan MAT 900, the VG 7070, VG ZAB-2F, Fisons AutoSpec, and Fisons ZabSpec, the JEOL SX-102A, HX-110/110, and JMS-700. Whatever the instrument, they were routinely equipped with EI ion sources, employed ion kinetic energies in the 3–10 kV range, and their high vacuum ion source housings were ready to accommodate FI/FD sources, typically developed and provided as optional equipment by the respective instrument manufacturers themselves.

When magnetic sector instruments began to vanish from the laboratories it was required to adapt FI/FD sources to other types of mass analyzers. Even though the adaptation to a linear quadrupole analyzer has been accomplished, it never really succeeded as the kinetic energy spread of ions emerging from the emitter was not well suited for this type of mass analyzer.^144–146^

The adaptation of FI, FD, and LIFDI to oaTOFs has been very successful and was commercialized by JEOL with the AccuTOF GC series^147–155^ and by Waters with the GCT series of instruments.^156–163^

As these oaTOF instruments, depending on the actual version of a particular instrument, provide a resolving power of 5000–10000, they are actually better suited for accurate mass measurements than scanning sector instruments. Nonetheless due to their temporal drift in mass calibration they are still limited in that respect.

The attachment of a LIFDI source to a Fourier transform-ion cyclotron resonance (FT-ICR) instrument combines LIFDI with ultimate resolving power and mass accuracy and was first realized by A. G. Marshall’s group.^59,60,164^ LIFDI-FT-ICR-MS was mostly employed for complex mixture analysis.^141,142,165–168^ Another LIFDI source has been constructed to work with Bruker ApexQe instruments.^61,62^ Unfortunately, the ion lifetime requirements in the order of seconds along with the comparatively low sensitivity of FT-ICR analyzers posed limitations to the success of this combination.

The next step was accomplished by the adaptation of LIFDI sources to Waters Q-TOF type of instruments that were originally designed for atmospheric pressure sources.^169–171^

**Figure 19.**
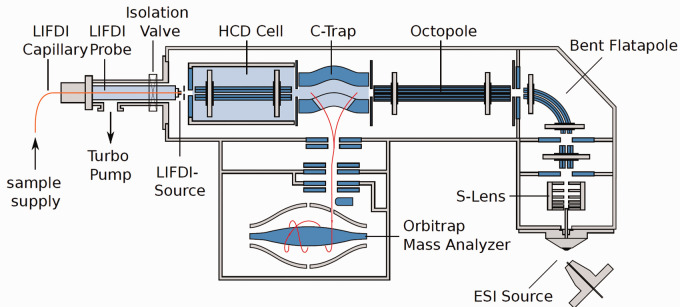
Adaptation of LIFDI to an Orbitrap via the HCD cell. The probe is inserted via a vacuum lock with a turbomolecular pump to meet the high vacuum requirements of the Orbitrap. This setup circumvents any interference with the atmospheric pressure source. Courtesy of Linden CMS, Leeste.

The attachment of LIFDI to the Orbitrap analyzer has taken a new route as this approach employs the HCD cell, essentially a linear octopole ion trap, as entry port for the LIFDI probe while it leaves the atmospheric pressure source on the opposite side of the instrument untouched (Figure 19). LIFDI-Orbitrap applications became quite numerous but while the results presented in these publications are heavily relying on accurate mass data by LIFDI-Orbitrap, they generally do not show these spectra.^172–179^

It is a general phenomenon that in the field of LIFDI-MS there are quite a number of custom solutions available that deliver good analytical results in routine use, while the exact setup of some of them has unfortunately never been published.

## Selected applications

There is a vast number of publications on developments and applications in FI, FD, and LIFDI. One important aspect is to show the results obtained by applying these techniques to different compound classes in order to demonstrate where they play to their strength. Another aspect is to provide an overview of the instrumental platforms and modes of operation (cf. preceding section). Sometimes, these aspects are interwoven, e.g., the attachment of LIFDI sources to FT-ICR instruments presents both an instrumental development and an application of LIFDI to complex mixture analysis at ultrahigh resolving power. To prevent this Account from reaching incommensurate length, this section is restricted to a selection of topics that should reflect the most relevant aspects.

### Hydrocarbon analysis by FI-MS and FD-MS

FI-MS presents a capable method to analyze volatile compounds and low to medium polarity samples that can at least be volatilized from a direct insertion probe. Some examples illustrating the softness of FI in comparison to EI as well as the general characteristics of FI-MS have already been presented in this Account (Figures 1, 3, 5–7). FI is not necessarily limited to compounds of low molecular weight but may cover analytes of up to about 800 u.

FI-MS has been used for the analysis of saturated and unsaturated aliphatic hydrocarbons, especially in the field of fuel analysis from early on.^7,11,50,180–187^ Applications on synthetic hydrocarbon polymers like polyethylene (PE) and polystyrene (PS) followed.^180,188–190^ The analysis of PE products could easily be performed for weight-average molecular weights in the range of 400–1000 u,^191,192^ while it becomes quite difficult to obtain FD-MS data of PE oligomers beyond 2000 u.^193^

FI spectra of aliphatic hydrocarbons tend to exhibit peaks corresponding to species with one or even two double bond equivalents more than expected. The occurrence of these additional peaks can be explained by fragmentation of the molecular ion via loss of one or two hydrogen molecules, respectively, to yield [M–H_2_]^+•^ and [M–2H_2_]^+•^ ions.

Studies on field-induced dehydrogenation of several pure hydrocarbons up to hexatriacontane, C_36_H_74_, and of polyethylene 655^194–196^ revealed that the degree of dehydrogenation is greatly reduced by applying lower electric fields and lower amounts of sample to the emitter. In other words, this type of reaction is bimolecular as higher particle density due to higher emitter load in FD leads to enhanced dehydrogenation.

**Figure 20.**
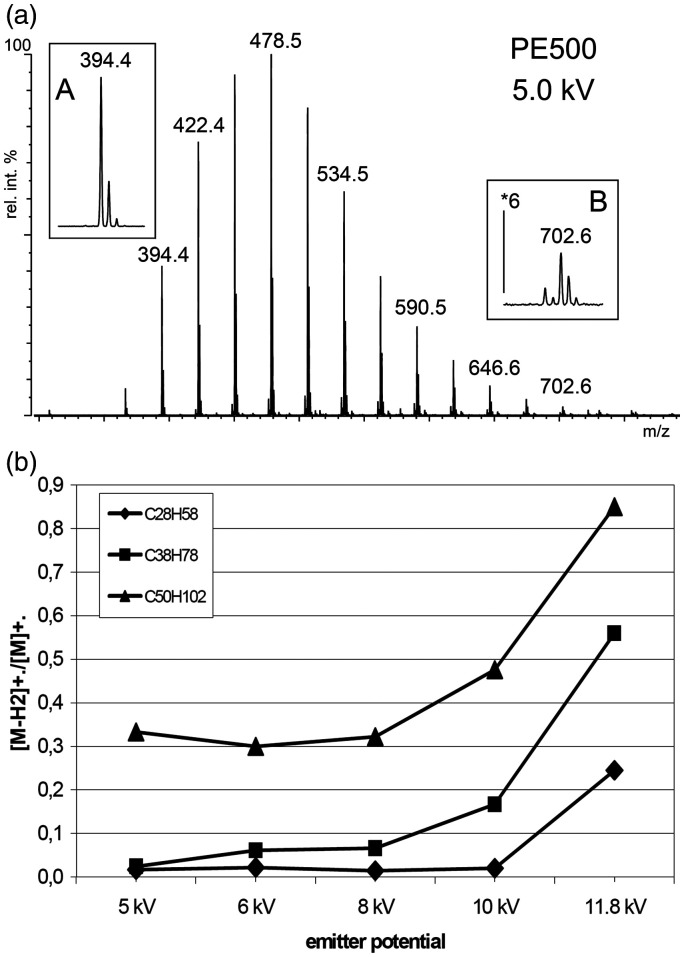
Hydrocarbon analysis by FD-MS. (a) The FD mass spectrum of polyethylene 500 at an emitter voltage of 5 kV shows no H_2_ loss from C_28_H_58_^+•^, *m/z* 394.4 (inset A), but notable loss from C_50_H_102_^+•^, *m/z* 702.6 (inset B). (b) The ratio of [M–H_2_]^+•^/M^+•^ increases along with the emitter potential. © Reproduced from Ref. [193] with kind permission. © SAGE Publishing, 2000.

With PE 500 and PE 655 series of spectra were recorded showing a strong influence of electric field strength (Figure 20).^193^ Among the oligomers in PE 500 at an emitter potential of 11.8 kV a [M-H_2_]^+•^/[M]^+•^ ratio of 0.83 was found for pentacontane, C_50_H_102_^+•^, *m/z* 702.8, that decreased to 0.33 at 5.0 kV. The effect was observed to be more pronounced for octatriacontane ions, C_38_H_78_^+•^, *m/z* 534.6, and octaeicosane ions, C_28_H_58_^+•^, *m/z* 394.5, were dehydrogenation almost disappeared at 5.0 kV. Generally, lower mass hydrocarbons were less affected by dehydrogenation. Analogous reactions were also observed in case of large multiply branched saturated hydrocarbons.^26^

**Figure 21.**
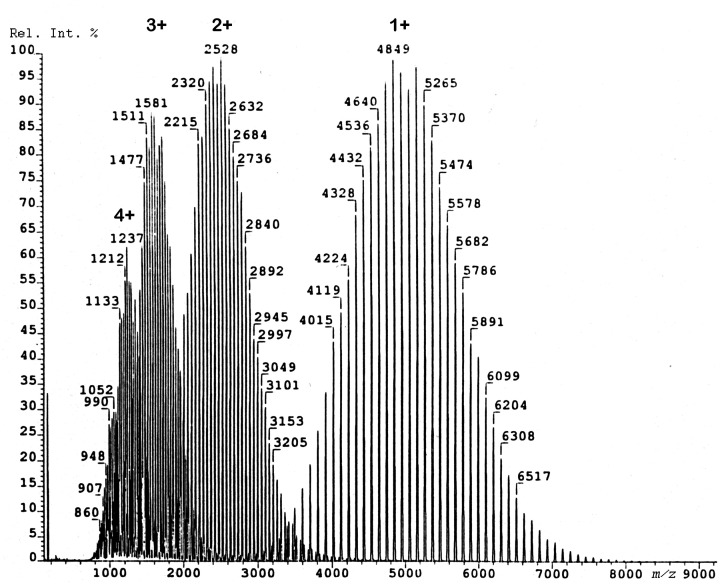
FD mass spectrum of a polystyrene sample of average molecular weight of 5100 u. In this case, the molecular weight distribution is not only represented by the singly charged molecular ions but also by partially overlapping series of doubly, triply, and even quadruple charged ions. Reproduced from Ref. [190] with kind permission. © John Wiley & Sons, 1990.

As an effect of its charge stabilizing aromatic systems PS forms series of M^+•^ ions, and therefore, PS is much easier to analyze across a larger mass range than PE.^197^ FD-MS analysis of PS has been demonstrated to work reasonably well up to about *m/z* 10,000.^189,190^ PS exceeding average molecular weights of 2000 u can also form ions of higher charge state (Figure 21). Moreover, PS serves as a mass calibrant in FD-MS as it provides well-spaced ion series at Δ(*m/z*) = 104 over a wide range.^154,189^

### FD and LIFDI for highly polar and ionic analytes

In FD – likewise in LIFDI – highly polar analytes are mainly forming [M+H]^+^ and [M+alkali]^+^ ions while molecular ions are normally absent or at least of very low abundance.^81,97,99–101,198–200^ The combination of high particle density at the emitter surface with the polarity of such molecules also promotes cluster ions like [*n*M+H]^+^ and [*n*M+alkali]^+^. Essentially, preformed ions are being desorbed by virtue of the electric field via ion desolvation^104–106^ or ion evaporation,^107,108^ respectively, because the field strength required for desorption of ions is below the threshold for field ionization.^86,100–103^

**Figure 22.**
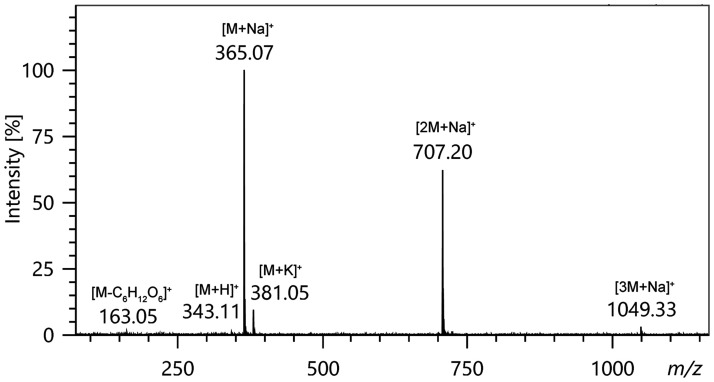
The LIFDI mass spectrum of saccharose mainly shows ions due to alkali adduct formation. Saccharose was applied as solution (ca. 1mg ml^−1^ in water: methanol = 1: 2) and the spectrum was acquired while ramping the EHC at 20 mA min^−1^. This spectrum is an experimental representation of the ion desolvation process as depicted in Figure 14.

**Example:** The LIFDI spectrum of saccharose, C_12_H_22_O_11_, *M*_w_ = 342.11 u, presents the typical behavior of oxygen-rich highly polar analytes in FD or LIFDI, respectively. Saccharose mainly forms [M+Na]^+^ ions, *m/z* 365.07, along with the cluster ions [2M+Na]^+^, *m/z* 707.20, and [3M+Na]^+^ at *m/z* 1049.33 (Figure 22). In addition, [M+H]^+^, *m/z* 343.11, and [M+K]^+^, *m/z* 381.05, ions are observed. Even though the disaccharide exhibits strong intermolecular bonding due to multiple hydrogen bridges, there is only a single fragment ion peak of very low intensity at *m/z* 163.05. For the most part, this fragmentation is thermally-induced as it mainly appears towards the end of the desorption process that started at an EHC of about 28 mA and ended at about 45 mA.

FD spectra of salts, CA, show the cation C^+^ along with less intensive peaks corresponding to the [C_2_A]^+^ and [C_3_A_2_]^+^ cluster ions. Occasionally even larger cluster ions may be detected. This behavior is independent of whether the salt cation is inorganic, organic or a metal complex.^23,27,30,32,36,201–203^

**Figure 23.**
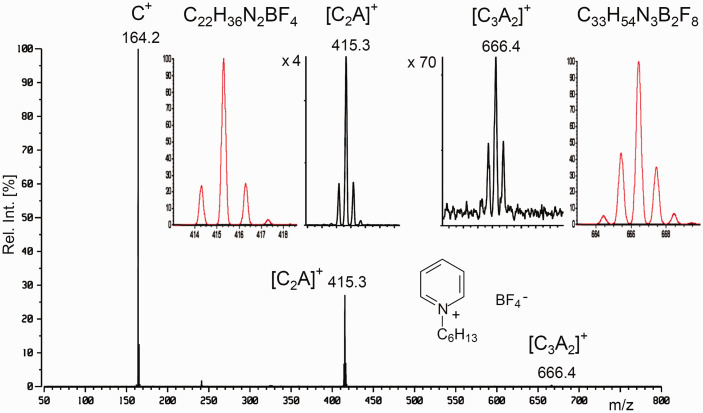
LIFDI mass spectrum of *N*-hexylpyridinium tetrafluoroborate in methanol at a concentration of 0.1 µl ml^−1^ scanned over the *m/z* 50–800 range. The insets show the [C_2_A]^+^ and [C_3_A_2_]^+^ cluster ions for comparison of the experimental and calculated isotopic patterns. Reproduced from Ref. [139] with kind permission. © American Chemical Society, 2007.

**Example:** The LIFDI spectrum of *N*-hexylpyridinium tetrafluoroborate presents a typical example for the appearance FD or LIFDI mass spectra of salts (Figure 23).^139^ The *N*-hexylpyridinium ion, C^+^, *m/z* 164.2, causes the base peak of the spectrum while the cluster ions [C_2_A]^+^, *m/z* 415.3, appear at 27% relative intensity and the [C_3_A_2_]^+^ cluster ions, *m/z* 666.4, at 1.3%. The insets show comparisons of the experimental and calculated isotopic patterns where the isotope ratio of ^10^B to ^11^B clearly indicates the presence of one boron atom at *m/z* 415.3 and two at *m/z* 666.4. Thus, the cluster ions allow for the identification of the anions, because the Δ(*m/z*) between C^+^ and [C_2_A]^+^ or between [C_2_A]^+^ and [C_3_A_2_]^+^ corresponds to [CA]. Subtracting the cation mass yields the anion mass and changes in the isotopic pattern from cation peak to cluster ion peak contain information on the elemental composition of the anion.

Of course, tandem mass spectrometry would be the tool of choice for the identification of cluster ions as they would easily undergo fragmentation to yield the next smaller cluster by loss of CA. However, a closer look at the temporal evolution of the ion currents corresponding to the species under investigation suffices to reveal their identity.

Ions of lower mass generally desorb at lower EHC than those of higher mass, i.e., there is some fractionation of components along the EHC ramp. In other words, in FD-MS of a mixture of cations (or any other analytes) of different molecular mass, the lightest will show up first and that of highest mass last. Cluster ions, however, behave the opposite way, because cluster ion formation is promoted by high particle density. As the emitter is heated and gets more and more depleted by desorption of ions the chances for cluster ion formation are diminishing. Therefore, larger cluster ions and higher cluster ion intensities are observed at the onset of ion desorption while C^+^ dominates towards the end of desorption.

**Figure 24.**
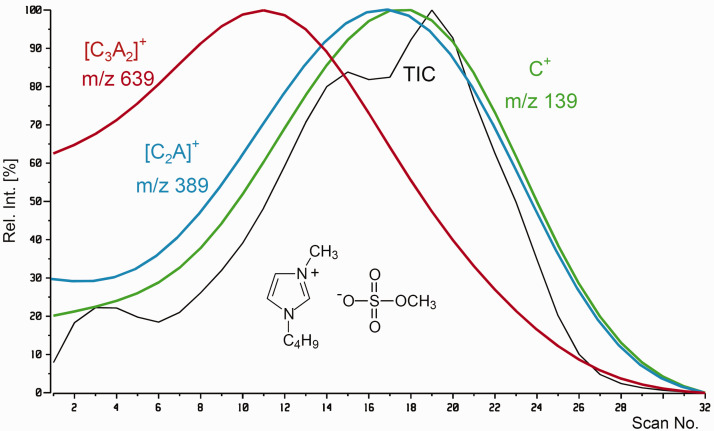
Total ion chromatogram (TIC) of the measurement of 1-butyl-3-methylimidazolium methylsulfate and reconstructed ion chromatograms (RICs) of C^+^, *m/z* 139, [C_2_A]^+^, *m/z* 389, and [C_3_A_2_]^+^, *m/z* 639. The largest cluster ion predominates at the beginning. Each curve is normalized to 100% relative intensity to simplify their comparison in time. Reproduced from Ref. [139] with kind permission. © American Chemical Society, 2007.

The LIFDI mass spectrum of 1-butyl-3-methylimidazolium methylsulfate shows the same characteristics as the example above and exemplifies the recognition of cluster ions by use of reconstructed ion chromatograms (RICs, Figure 24). The figure compares the total ion chromatogram (TIC) and the RICs of C^+^, *m/z* 139, [C_2_A]^+^, *m/z* 389, and [C_3_A_2_]^+^, *m/z* 639. The largest cluster ion dominates at the onset of desorption while the cation peak exhibits the highest relative intensity closer to the end.

### Applications of LIFDI-MS

LIFDI has been introduced with the intention of enabling the application of FD to compounds sensitive to atmospheric components, in particular oxygen, carbon dioxide, and water.^56^ In fact, the majority of publications mentioning LIFDI deals with compounds such as reactive transition metal complexes and catalysts.^56,138,204–217^ Another group belongs to the field of petroleomics applications as LIFDI turned out to be compatible to FT-ICR instrumentation.^59,140–142,164,218^ As LIFDI also serves as a general replacement for FD, there are miscellaneous applications in addition.^61,62,139,155,167^ As already mentioned in the section on mass analyzers for FI, FD, and LIFDI, numerous publications depend on LIFDI data but do not discuss or even show these spectra.

**Figure 25.**
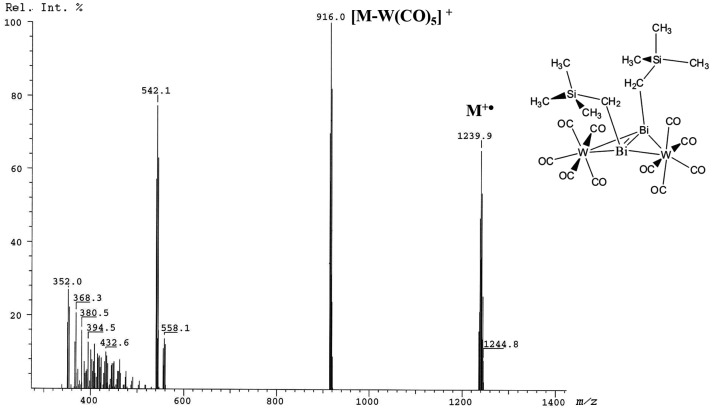
LIFDI spectrum of the complex [μ-η^2^-(*cis*-Me_3_SiCH_2_Bi)_2_][W(CO)_5_]_2_ as obtained due to inert conditions. Reproduced from Ref. [56] with kind permission. © SAGE Publishing, 2004.

The LIFDI spectrum of the dibismuthene tungsten carbonyl complex [μ-η^2^-(*cis*-Me_3_SiCH_2_Bi)_2_][W(CO)_5_]_2_ presents one of the earliest examples where LIFDI ensures the required degree of inert sample deposition (Figure 25).^56^ The sample was dissolved in dry toluene and stored under argon atmosphere until the measurement was performed on a Finnigan MAT-900 double focusing magnetic sector instrument. While the fragment ion due to loss of one W(CO)_5_ moiety gave rise to the base peak, *m/z* 916.0, the intensive molecular ion signal, *m/z* 1239.9, exhibited an isotopic pattern in very good agreement with the calculated pattern.

**Figure 26.**
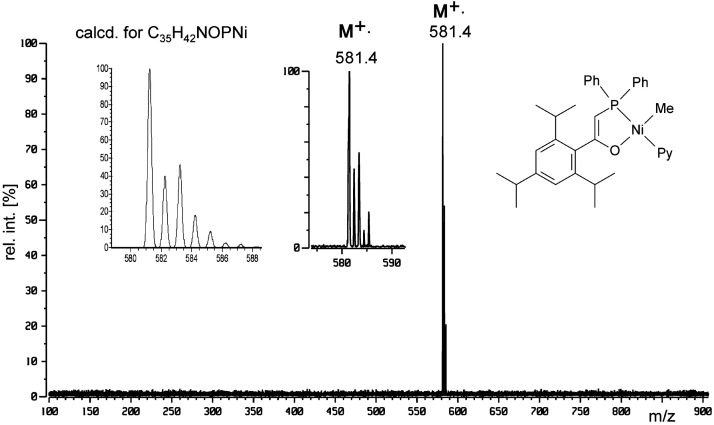
LIFDI mass spectrum of a Ni complex from solution in toluene showing no fragmentation. The spectrum was measured on a JEOL JMS-700 double focusing sector instrument. The observed isotopic pattern of M^+•^ corresponds to the calculated isotopic distribution (cf. insets). Reproduced from Ref. [138] with kind permission. © Springer Nature, 2006.

Both extraordinary softness of FD and inertness of sample handling provided by LIFDI are exemplified by the LIFDI spectrum of a complex with a Ni–pyridine and even a Ni–Me bond. The LIFDI mass spectrum of [1-diphenylphosphino-2-(2′,4′,6′-triisopropylphenyl)-ethene-2-olate-κ^2^*O,P*]nickel(II)(methyl)(pyridine) exclusively exhibits the molecular ion at *m/z* 581.4 (Figure 26).^138^ Again, experimental and calculated isotopic pattern were found in good agreement.

### Accurate mass measurements

The value of high-resolution and accurate mass data has generally been recognized in the MS community. In fact, accurate mass FI data has already been obtained in the 1970s.^18,19,134,219^ Due to the low ion currents it is not trivial to achieve high resolution while maintaining a well-defined peak shape and good signal-to-noise ratio to deliver the mass accuracy required for formula assignment. Another problem is related to the fact that common mass calibrants like perfluorokerosine (PFK) and perfluorotributylamine (PFTBA aka FC43) exhibit very low ionization efficiency in FI, thereby limiting their application as internal calibrants.^136^ Apart from this, in FI, PFK and PFTBA only cover the low-mass range up to about *m/z* 600. In FD-MS the mass calibrant ideally should desorb synchronous with the analyte or at least shortly before or after its desorption as an increased time span between calibrant and analyte tends to lower the mass accuracy. This is particularly relevant when analyzer calibrations tend to vary from scan to scan like magnetic sectors do^220^ or tend to drift in time like TOFs do.

The best option to realize high-resolution accurate mass measurements in FI, FD or LIFDI mode is to combine them with mass analyzers that do not require internal calibration, which is the case with FT-ICR^59–62,141,142,164–168^ and Orbitrap systems.^172–179^

### Gas chromatography-field ionization-mass spectrometry

Gas chromatography-field ionization (GC-FI) is the easiest to set up hyphenation within the FI/FD/LIFDI family, because for the most part, it just means doing FI with sample introduction from a gas chromatograph, and therefore has been accomplished soon after the introduction of FI-MS.^221–223^

In GC-FI, all parameters of the gas chromatographic separation that may have been established in EI mode can be used without any modification. Differences to EI are caused by much lower ion currents, and consequently, the strong need to optimize the ion yield. Typical measures to do so include the use of dedicated FI emitters and to adjust the GC capillary exit in optimum position with respect to the emitter in order to get as much as possible of the eluate into the zone of ionization. The fact that the helium carrier gas is not ionized by FI due to its high ionization energy of 24.6 eV actually presents an advantage for GC-FI operation.^80^

The combination of GC-FI and low ion currents of FI turned out to be limiting for the combination thereof with the slow-scanning magnetic sector instruments. The advent of orthogonal-acceleration time-of-flight (oaTOF) instruments with their high duty cycle^224,225^ has changed the situation.^57,58,84,143,226^ Thus, GC-FI-oaTOF-MS became established in several laboratories, in particular for petroleum analysis.^57,58,227,228^

In contrast to FI-MS of single compounds, GC-FI requires continuous operation of the emitter for several tens of minutes. Elongated sample supply, however, causes a gradual decrease of the activity of the emitter due to the formation of surface layers. To maintain the emitter activity, it is usually baked, a procedure that is difficult to perform during acquisition. The problem can be solved by flash-heating the emitter for 0.02–0.10 s after an accumulation period of 0.5–1.0 s, i.e., once after saving a spectrum to disk.^57,58^ Employing acquisition-synchronized emitter flash heating enables GC-FI operation for hours. GC-FI has also been driving the development of EI/FI/FD^75,226^ and EI/CI/FI combination sources for GC-oaTOF instruments.^84^

Recent work impressively demonstrates the usefulness of FI in conjunction with two-dimensional gas chromatography (GCxGC) for complex mixture analysis. Applications of GCxGC-FI to hydrocarbon fuels dominate^229–233^ but the method also serves for biomarker analysis.^234^ GC-FI-MS is frequently employed to complement GC-EI-MS data by enabling a reliable assignment of the molecular mass of unknowns what can be difficult to achieve when molecular ion peaks are of very low intensity or even absent in EI spectra.

**Figure 27.**
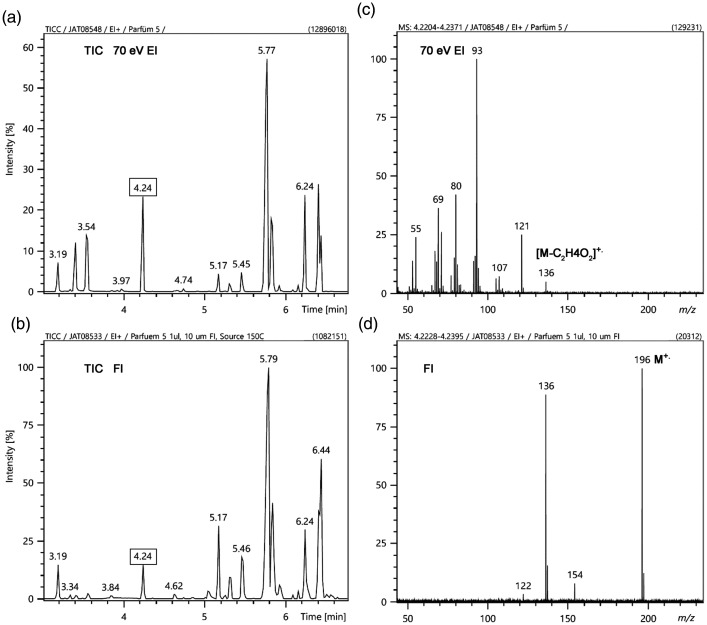
GC-MS analysis of ck one eau de toilette (1 µL injected at 1: 30 split ratio on 25-m HP-5 column, 50°C for 1 min, to 300°C at 35°C min^−1^). (a) TIC by GC-EI, (b) TIC by GC-FI, (c) EI mass spectrum of the compound eluting at 4.24 min, and (d) FI mass spectrum of the same compound.

**Example:** The eau de toilette product ck one by Calvin Klein has been analyzed by both GC-EI-MS and GC-FI-MS to explore the GC-FI functionality and acquisition-synchronized emitter flash heating of a new type of LIFDI ion source.^154^ No special attempt was made to achieve full separation of all compounds. While TICs in both modes showed the same number of peaks, their relative intensities varied due to different ionization efficiencies of the components in EI versus FI mode (Figure 27). The spectra of the component eluting at 4.24 min may illustrate the advantage of FI for molecular ion recognition. As indicated by searching the NIST/EPA/NIH mass spectral data base 2017, the EI spectrum corresponds to linalyl acetate, C_12_H_20_O_2_, *M*_w_ = 196 u. The EI spectrum, however, does not show a molecular ion peak and the ion at *m/z* 136 should thus reflect a [M–C_2_H_4_O_2_]^+•^ fragment. It is therefore reasonable to obtain confirmation of the molecular mass by referring to the FI spectrum that exhibits the M^+•^ ion, *m/z* 196, as the base peak plus the indicative [M–C_2_H_4_O_2_]^+•^ ion, *m/z* 136.

### Continuous-flow LIFDI

**Figure 28.**
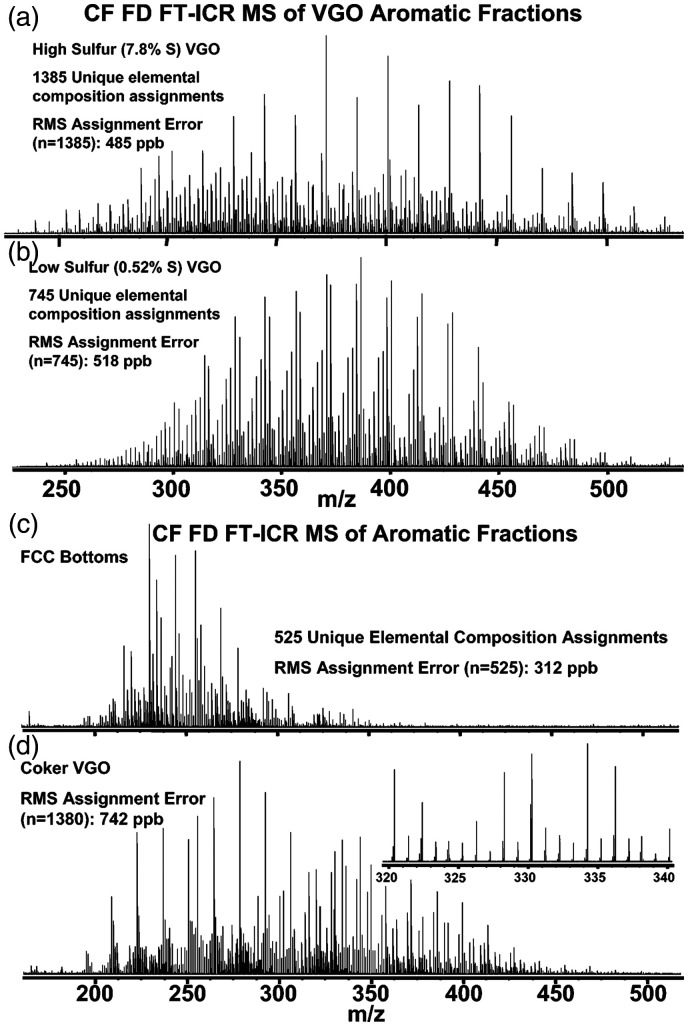
Broad-band continuous-flow LIFDI-FT-ICR mass spectra of aromatic fractions. (a) A high-sulfur vacuum gas oil and (b) a low-sulfur vacuum gas oil. (c) Catalytic cracking bottoms and (d) a coker vacuum gas oil. These spectra result from co-addition of 75 time-domain signals, each based on 20 s of external LIFDI ion accumulation. The total analysis period per final spectrum was approximately 1 h. Reproduced from Ref. [166] with kind permission. © American Chemical Society, 2005.

Continuous-flow (CF) LIFDI presents a precursor stage to hyphenation of liquid chromatography with LIFDI. The introduction of CF-LIFDI was meant to provide elongated sample supply as required to analyze extremely complex oil fractions where each component was only present at a very low concentration. The number of ions generated per conventional emitter load turned out to be too low to acquire a useful spectrum on a Fourier transform-ion cyclotron resonance (FT-ICR) mass spectrometer, the resolving power of which was necessary to separate as many components as possible. CF-LIFDI was realized by admitting the sample solution (0.1 mg ml^−1^) at 75 nl min^−1^ through a 10 µm i.d. capillary from a syringe pump while the ionizing high voltage was applied to the emitter. Normally, the high voltage needs to be switched off during sample supply as ionization of the solvent would cause an electric discharge and thus emitter rupture. The very low solvent flow plus slight heating of the emitter at 15 mA allowed to deal with the solvent without detrimental side effects.^60,166,235^ In a typical CF-LIFDI experiment, 50–75 transients, each based on an ion population collected from 20 s of ion emission, were accumulated to achieve the desired level of mass resolving power and signal-to-noise ratio also permitting formula assignment for components of low abundance (Figure 28). By CF-LIFDI an improved spectral quality was achieved as the ion current could be sustained for data accumulation totaling of up to 1 h.

## Comparison to other ionization methods

At last, the properties of FI, FD, and LIFDI should briefly be compared to other (soft) ionization methods (Table 3). As mentioned in the Historical Sketch, FAB, another desorption/ionization method, appeared in the early 1980s and soon became a strong competitor to FD.^40,41^ Like FD, FAB can deal with neutral and ionic analytes as it is capable of ionizing molecules via various pathways to yield either molecular ions, M^+•^, or adduct ions by protonation or cationization, i.e., [M+H]^+^, [M+NH_4_]^+^, [M+alkali]^+^. As an advantage over FD, FAB can easily be switched to negative-ion mode. Although versatile and convenient to use, FAB disappeared from the laboratories along with the magnetic sector instruments, and thus, does not play a role anymore.

**Table 3.** FI, FD, and LIFDI in comparison to other ionization methods.

Polarity	FI	FD	LIFDI	EI	APCI	FAB	DART	ESI	MALDI
Positive ions	+	+	+	+	+	+	+	+	+
Negative ions	–	(+)^1^	–	–	+	+	+	+	+
Analyte property									
Nonpolar M	+	+	+	+	(+)	+	(+)	–	(+)
Polar M	+	+	+	+	+	+	+	(+)	+
Highly polar M	(+)	+	+	(+)	+	+	+	+	+
Ionic CA	–	+	+	–	(+)	+	+	+	+
Volatile	+	–	–	+	+	–	+	–	–
Involatile	–	+	+	–	+	+	+	+	+
Air/water sensitive	+	–	+	(+)^2^	–	(+)^2^	–	(+)^3^	–
Other									
GC-MS	+	–	(+)^4^	+	(+)^5^	–	–	–	–

^1^Only selected instruments can do; rarely used.

^2^Special sample handling like glove box required.

^3^Restricted to ionic analytes in dry aprotic solvents.

^4^When LIFDI probe is used in FI mode.

^5^Special GC interface required.

MALDI^45,46,236–238^ can be seen as the successor to FAB as both are relying on a matrix to embed sample molecules before energy is impacted to the sample layer. The use of light as the primary source of energy causes MALDI to exhibit some differences to FAB, often rendering MALDI softer than FAB, although in some cases like transition metal carbonyl complexes FAB may be softer than MALDI, while FD is still softer than either of them.^25^ ESI^43,44,236^ certainly presents the softest technique to transfer ions from solution into the gas phase. In contrast to FD, FAB and MALDI, however, ESI can only deal with analytes that are ionic or are readily forming ions in solution, e.g., by protonation or cationization. ESI does not provide a pathway of ionization that would allow to deal with neutral analytes having no heteroatoms, in other words, molecular ion formation is excluded. Like FAB and MALDI, ESI can be used in both positive-ion and negative-ion mode.

Direct analysis in real time (DART), one of the ambient desorption/ionization techniques,^239–242^ resembles atmospheric pressure chemical ionization (APCI) in terms of how analyte molecules are ionized.^243,244^ The coverage of analytes by DART (and APCI) is quite similar to FAB and MALDI. Different from LIFDI, neither of these methods is suited to allow for sample handling under inert conditions. Thus, FI, FD, and LIFDI are particularly useful when softness of ionization is very important, when analytes are neutral molecules, especially those of low polarity, or when inert sample admission is required.

## Conclusion and perspective

The techniques described in this Account are not brand new but they are mature! The journey began with the discovery of FI in the 1950s and has continued until today. The success of FI, FD, and later LIFDI was and still is much based on the extraordinary softness of ionization and on the wide range of analytes they are able to deal with. More recent ionization methods like ESI, MALDI, and the numerous approaches to ambient ionization obviously are governing modern mass spectrometry. Nonetheless, being able to ionize compounds without any heteroatom or even without any functional group, working without any need for matrix or dopant, and allowing even highly reactive analytes to be transferred into the ion source without decomposition represents something special. FI and FD have seen their ups and downs and might have been superseded if LIFDI had not been introduced. The advent of LIFDI completed a family of ionization methods and initiated an ongoing development that aims at adapting the technique to more types of mass analyzers and also opens the door to hyphenation. GC-FI is established, CF-LIFDI could provide an entry to LC coupling, and time will tell what is to come. The decade ahead of us will tell.
